# Cholesterol Efflux Capacity and Its Association With Adverse Cardiovascular Events: A Systematic Review and Meta-Analysis

**DOI:** 10.3389/fcvm.2021.774418

**Published:** 2021-12-13

**Authors:** Jane J. Lee, Gerald Chi, Clara Fitzgerald, Syed Hassan A. Kazmi, Arzu Kalayci, Serge Korjian, Danielle Duffy, Alka Shaunik, Bronwyn Kingwell, Robert W. Yeh, Deepak L. Bhatt, C. Michael Gibson

**Affiliations:** ^1^Baim Institute for Clinical Research, Boston, MA, United States; ^2^Beth Israel Deaconess Medical Center, Harvard Medical School, Boston, MA, United States; ^3^CSL Behring, King of Prussia, PA, United States; ^4^CSL Behring, Bio21, Parkville, MO, Australia; ^5^Smith Center for Outcomes Research in Cardiology, Beth Israel Deaconess Medical Center, Harvard Medical School, Boston, MA, United States; ^6^Brigham and Women's Hospital, Harvard Medical School, Boston, MA, United States

**Keywords:** acute coronary syndrome, atherosclerosis, acute myocardial infarction, cholesterol, cholesterol efflux capacity (CEC)

## Abstract

**Background:** Serum high-density lipoprotein cholesterol (HDL-C) levels are inversely associated with cardiovascular disease events. Yet, emerging evidence suggests that it is the functional properties of HDL, in particular, reverse cholesterol transport, which is a key protective mechanism mediating cholesterol removal from macrophage cells and reducing plaque lipid content. Cholesterol efflux capacity (CEC) measures the capacity of HDL to perform this function. A systematic review and meta-analysis were conducted to explore the association of CEC and adverse cardiovascular events.

**Methods:** A comprehensive literature review of Embase, PubMed, and Web of Science Core Collection from inception to September 2019 was performed for all studies that examined the association between CEC and cardiovascular outcomes. The primary outcome was adverse cardiovascular events, which were inclusive of atherosclerotic cardiovascular disease (ASCVD) or mortality.

**Results:** A total of 20 trials were included. Compared with low CEC levels, high CEC levels were associated with a 37% lower risk of adverse cardiovascular events (crude RR = 0.63; 95% CI, 0.52–0.76; *P* < 0.00001). Every SD increase of CEC was associated with a 20% lower risk of adverse cardiovascular events (HR = 0.80; 95% CI, 0.66–0.97; P = 0.02). The association remained significant after adjusting for cardiovascular risk factors, medications, and HDL-C levels (HR = 0.76; 95% CI, 0.63–0.91; *P* = 0.004). A significant CEC-endpoint relationship was observed (*P* = 0.024) such that for every 0.1 unit increase in CEC, there was a 5% reduced risk for adverse cardiovascular events (RR = 0.95; 95% CI, 0.91–0.99).

**Conclusions:** Higher CEC is associated with lower adverse cardiovascular outcomes. These findings warrant further research on whether CEC is merely a biomarker or a mechanism that could be targeted as a pharmacologic intervention for improving clinical outcomes.

**PROSPERO Registration Number:** CRD42020146681; https://www.crd.york.ac.uk/prospero/.

## Introduction

An inverse relationship between high-density lipoprotein cholesterol (HDL-C) concentration and atherosclerotic cardiovascular disease (ASCVD) has been established through numerous observational studies and clinical trials (1, 2). However, the mechanisms underlying this association are not completely understood. Pharmacological studies have challenged the hypothesis that increasing levels of HDL-C would decrease ASCVD risk (3, 4). Mendelian randomization studies have demonstrated that genetic variants associated with high HDL-C levels were not associated with low ASCVD risk (5–7). Rather than crude HDL-C concentrations, emerging evidence has suggested that a quantitative measure of HDL functionality may be a better predictor of ASCVD risk.

A key mechanism by which HDL mitigates the development of atherosclerosis is through reverse cholesterol transport, which promotes cholesterol efflux from macrophages within atherosclerotic plaques. HDL functions to transport excess cholesterol to the liver, thereby reducing the formation of foam cells, which is a key component of atherosclerosis development. Cholesterol efflux capacity (CEC) measures the ability of HDL to promote cholesterol efflux from macrophages, the first step in reverse cholesterol transport. Greater CEC or improved HDL function, rather than higher HDL-C concentrations, is hypothesized to be a mechanism of ASCVD risk reduction.

Recent literature has shed light on the association between increased CEC and decreased ASCVD risk in the outpatient setting. Most notably, the Dallas Heart Study reported an inverse relationship between CEC and incident cardiovascular events, and the EPIC-Norfolk study found consistent results using a nested case-control design (8, 9). The present study aimed to review and synthesize the current evidence regarding the association between CEC and adverse cardiovascular events.

## Methods

### Search Strategies and Selection Criteria

Systematic literature searches were performed in Embase, PubMed, and Web of Science Core Collection. The searches included a set of keywords, wildcards, truncation and medical subject headings, including cholesterol efflux capacity, atherosclerotic cardiovascular disease, atherosclerosis, coronary artery disease (CAD), acute coronary syndrome, myocardial infarction, stroke, cerebrovascular event, mortality, and death. The search terms were organized in thematic building blocks that could be combined as required. Human studies, published as original research articles, letters, or abstracts, that reported measurement of cholesterol efflux capacity at baseline as well as adverse cardiovascular events, including ASCVD or mortality were included. All searches were limited to English language and the time from inception to September 2019. Duplicates were removed before screening references. Detailed queries are provided in Supplementary Table S1.

### Data Extraction

Data extracted from each study included baseline characteristics, methods for CEC measurement, CEC levels, and frequencies or risk estimates for adverse cardiovascular events. Database search, article screening, and study selection were performed independently by two investigators using a standardized approach. Disagreement in extracted data was adjudicated by a third investigator. A flow diagram depicting the process of literature search and screening is provided in Supplementary Figure S1.

### Quality Assessment

Two independent investigators assessed the quality of case-control studies and cohort studies in accordance with the Newcastle–Ottawa Scale. Disagreement in the quality assessment was resolved by discussion and consensus. The quality assessment criteria and forms are provided in Supplementary Tables S2–S5.

### Study Endpoints and CEC Measurements

The primary endpoint is adverse cardiovascular events, defined as the composite of ASCVD or all-cause mortality. ASCVD was inclusive of acute coronary syndrome, stroke, arterial revascularization, atherosclerotic plaque (including coronary carotid and femoral atherosclerotic detected by angiography or ultrasonography), and cardiovascular death. Death from all causes and death from cardiovascular causes were also evaluated.

Global CEC was captured from each study for assessment of association with adverse cardiovascular events. Normalized CEC levels (expressed as arbitrary units [AU]) in reference to the CEC of serum controls were used in assessing the strength of the CEC-endpoint relationships. To document the methodological variability of quantifying CEC, information regarding the type of cholesterol donor cell (mouse macrophage cell line [J774] or human macrophage cell line [THP-1 macrophages] and cholesterol tracer were extracted from each study.

### Statistical Analysis

Several approaches were deployed to investigate the relationship between CEC and endpoints (including adverse cardiovascular events, ASCVD, death from all causes, and death from cardiovascular causes). First, the relative risk (RR) of high CEC group vs. low CEC group was examined. High CEC group was defined as the group above the median CEC or the top quartile or tertile (i.e., better CEC), whereas the low CEC group was the group below the median CEC or the bottom quartile or tertile (i.e., worse CEC). Second, the risk of outcomes associated with each standard deviation (SD) increment of CEC was assessed. Third, the strength of CEC-endpoint relationships were explored using the *dosresmeta* package in R. In brief, the relationship between the log-transformed CEC and endpoint for each study was estimated by fitting a linear regression model based on the number of cases and controls as well as cohort size from at least three quantitative exposure categories. The generalized least squares method was applied to estimate the covariances and the vector of the regression coefficients. The CEC concentration assigned to each level of functionality category was approximated from the mean or median as reported by the studies. Pooled RR with Wald-type confidence interval (CI) associated with every 0.1 unit increase in CEC was calculated. Subsequently, to test the potential non-linear association, a restricted cubic spline model was constructed, with three knots located at 10th, 50th, and 90th percentiles of the aggregated exposure distribution. Non-linearity was assessed under the null hypothesis that the coefficient of the second spline (i.e., between 10th and 50th percentiles) was equal to zero. The two regression coefficients and the variance-covariance matrix within each study were then combined in a random-effects meta-analysis. Last, a separate analysis was performed among the case-control studies to compare the mean CEC level between cases (individuals with adverse cardiovascular events, with ASCVD, or died) and controls (individuals without adverse cardiovascular events, without ASCVD, or survived).

Measures of effect included relative risk (RR), odds ratio (OR), and hazard ratio (HR), with or without adjustment as reported by the studies. The DerSimonian-Laird random-effects model was fitted to derive the combined overall estimate of the treatment effects. Heterogeneity among the studies was evaluated using Cochran's Q test (with the threshold of *P* > 0.10) and Higgins's I^2^ statistic (with the values of 0.25, 0.50, and 0.75 indicating a low, moderate, and high degree of heterogeneity, respectively).

Contour-enhanced funnel plots (with a significance level of 1, 5, and 10%) and Egger's test were employed to detect small-study effects for the endpoints with a study number of ten or more. The trim-and-fill method was used to adjust for publication bias. Subgroup analysis was performed to examine the robustness of the association among three subsets: (1) individuals without cardiovascular risk factors or chronic kidney disease (CKD); (2) individuals with cardiovascular risk factors (e.g., with underlying or a history of CAD, dyslipidemia, family history of myocardial infarction); and (3) individuals with CKD (e.g., estimated glomerular filtration rate [eGFR] <90 ml/min/1.73 m^2^, patients on dialysis, or renal transplant recipients). All analysis was performed using R software (Version 3.5.2; The R Foundation for Statistical Computing), Review Manager (Version 5.3; The Nordic Cochrane Center, The Cochrane Collaboration), Stata (StataCorp. 2019. Stata Statistical Software: Release 16. College Station, TX: StataCorp LLC), and SAS (Version 9.4; SAS Institute Inc.).

## Results

A total of 25,132 subjects from 20 studies were included in the meta-analysis and summarized in Table 1 (8–27). Ten studies included individuals with cardiovascular risk factors, four studies included patients with CKD, and six studies included general populations without cardiovascular risk factors or CKD. The mean age ranged from 42 to 69 years. The proportion of males ranged from 26.4 to 100%. The follow-up duration varied from 1 to 16 years. Three and 17 studies used THP-1 (human) and J774 (mouse) as the macrophage cell type donating labeled cholesterol in the CEC assay, respectively. Five studies measured the efflux of a fluorescent sterol (BODIPY-cholesterol), whereas 15 used radioisotope labeling ([^3^H]-cholesterol) in the CEC assays. The quality of the studies was generally high, with scores ranging from 5 to 9, as evaluated with the Newcastle-Ottawa Scale (Supplementary Tables S2–S5).

**Table 1:** Summary of included studies.

**Author (Year)**	**Study Design**	**Population**	**N**	**Mean Age**	**Male**	**Follow-Up**	**Endpoints [number of events/cases]**	**Donor Cell**	**Labeling**	**NOS**
Ebtehaj et al. (10)	Case-control; prospective	General population	705	59.0	71.2%	12 years	ASCVD (cardiovascular death and hospitalization for MI/coronary revascularization) [351]	J774	[^3^H]-Cholesterol	8
Cahill et al. (11)	Case-control; prospective	General population	1,397	63.0	100%	16 years	ASCVD (nonfatal MI and fatal CHD) [701]	J774	[^3^H]-Cholesterol	8
Guerin et al. (12)	Cohort; prospective	Patients with acute MI	1,609	63.0	75.7%	1.9 years	Death (all-cause mortality) [239]	THP-1	[^3^H]-Cholesterol	7
Chindhy et al. (13)	Cohort; prospective	CKD vs non-CKD patients	2,805	NR	NR	11.3 years	ASCVD (nonfatal MI, stroke, cardiovascular death) [131]; CVD [187]	J774	BODIPY-Cholesterol	8
Tejera-Segura et al. (14)	Case-control; cross-sectional	RA patients	401	57.2	26.4%	N/A	ASCVD (presence of atherosclerotic plaque in carotid artery) [66]	J774	BODIPY-Cholesterol	6
Khera et al. (15)	Case-control; prospective	Individuals with LDL-C <130 mg/dL and hsCRP ≥2.0 mg/L	1,050	69	71.6%	1.9 years	ASCVD (MI, hospitalization for unstable angina, arterial revascularization, stroke, or cardiovascular death) [314]	J774	[^3^H]-Cholesterol	8
Bauer et al. (16)	Cohort; prospective	CKD patients	526	65	59%	4.6 years	ASCVD (MI, arterial revascularization, stroke, lower extremity amputation, or cardiovascular death) [114]	J774	[^3^H]-Cholesterol	7
Gall et al. (17)	Case-control; cross-sectional	Patients with dyslipidemia	1,202	56.4	51.3%	N/A	ASCVD (presence of atherosclerotic plaque in carotid [7] or femoral artery [72])	J774	[^3^H]-Cholesterol	8
Kopecky et al. (18)	Cohort; prospective	Patients with T2DM on hemodialysis	1,147	66.3	54.8%	4.1 years	ASCVD (cardiovascular death, nonfatal MI, and stroke) [423]; Death [561]	THP-1	[^3^H]-Cholesterol	7
Javaheri et al. (19)	Cohort; prospective	Cardiac transplant recipients with CAV	35	44.4	85.7%	1 year	Death (all-cause mortality) [15]	J774	[^3^H]-Cholesterol	7
Mody et al. (20)	Cohort; prospective	General population and a subgroup with FHx of MI	1,972	44.9	44%	9.4 years	ASCVD (nonfatal MI, nonfatal stroke, coronary revascularization, or cardiovascular death) [97]	J774	BODIPY-Cholesterol	9
Liu et al. (21)	Cohort; prospective	Patients with CAD	1,737	63.5	65.2%	3.8 years	Death (all-cause mortality) [166]; CVD (cardiovascular death) [122]	J774	BODIPY-Cholesterol	9
Zhang et al. (22)	Cohort; prospective	Patients with SAP or ACS	429	66.2	74.8%	3 years	ACS [214]; ASCVD (nonfatal MI, nonfatal stroke, or cardiovascular death) [34]; CVD [22]	J774	[^3^H]-Cholesterol	8
Ogura et al. (23)	Cohort; cross-sectional	Patients with HeFH	227	57	44.5%	N/A	ASCVD (MI, stroke, angina pectoris with significant stenosis >75% on coronary angiogram, and coronary revascularization) [76]	J774	[^3^H]-Cholesterol	5
Annema et al. (24)	Cohort; prospective	Renal transplant recipients	495	51.6	54.3%	7.0 years	Death [102]; CVD [54]	THP-1	[^3^H]-Cholesterol	8
Ishikawa et al. (25)	Case-control; cross-sectional	Patients with suspected CAD	254	65.7	78.0%	N/A	ASCVD (native coronary atherosclerosis with >50% stenosis) [182]	J774	[^3^H]-Cholesterol	8
Saleheen et al. (9)	Case-control; prospective	General population	3,494	65.5	64.5%	12 to 16 years	ASCVD (unstable angina, stable angina, and fatal/nonfatal MI) [1745]	J774	[^3^H]-Cholesterol	7
Rohatgi et al. (8)	Cohort; prospective	General population	2,924	42	43%	9.4 years	ASCVD (nonfatal MI, stroke, cardiovascular death, and coronary revascularization) [132]; CVD (cardiovascular death) [42]	J774	BODIPY-Cholesterol	9
Li et al. (26)	Case-control; cross-sectional	General population (angiography & outpatient cohort)	1,727	60.6	54.1%	N/A	ASCVD (MI/CAD/coronary revascularization) [1017]	J774	[^3^H]-Cholesterol	7
Khera et al. (27)	Cohort; cross-sectional	Patients with CAD and controls	996	57.5	58.5%	N/A	ASCVD (angiographically confirmed coronary artery disease with >50% stenosis in a major coronary vessel) [442]	J774	[^3^H]-Cholesterol	7

*ACS, acute coronary syndrome; ASCVD, atherosclerotic cardiovascular disease; CAD, coronary artery disease; CAV, cardiac allograft vasculopathy; CHD, coronary heart disease; CKD, chronic kidney disease; CVD, cardiovascular death; FHx, family history; HeFH, heterozygous familial hypercholesterolemia; hsCRP, high-sensitivity C-reactive protein; LDL-C; low-density lipoprotein cholesterol; MI, myocardial infarction; N/A, not applicable; NOS, Newcastle–Ottawa Scale; NR, not reported; RA, rheumatoid arthritis; SAP, stable angina pectoris; T2DM, type 2 diabetes mellitus*.

### Association With Adverse Cardiovascular Events

**Figure 1:**
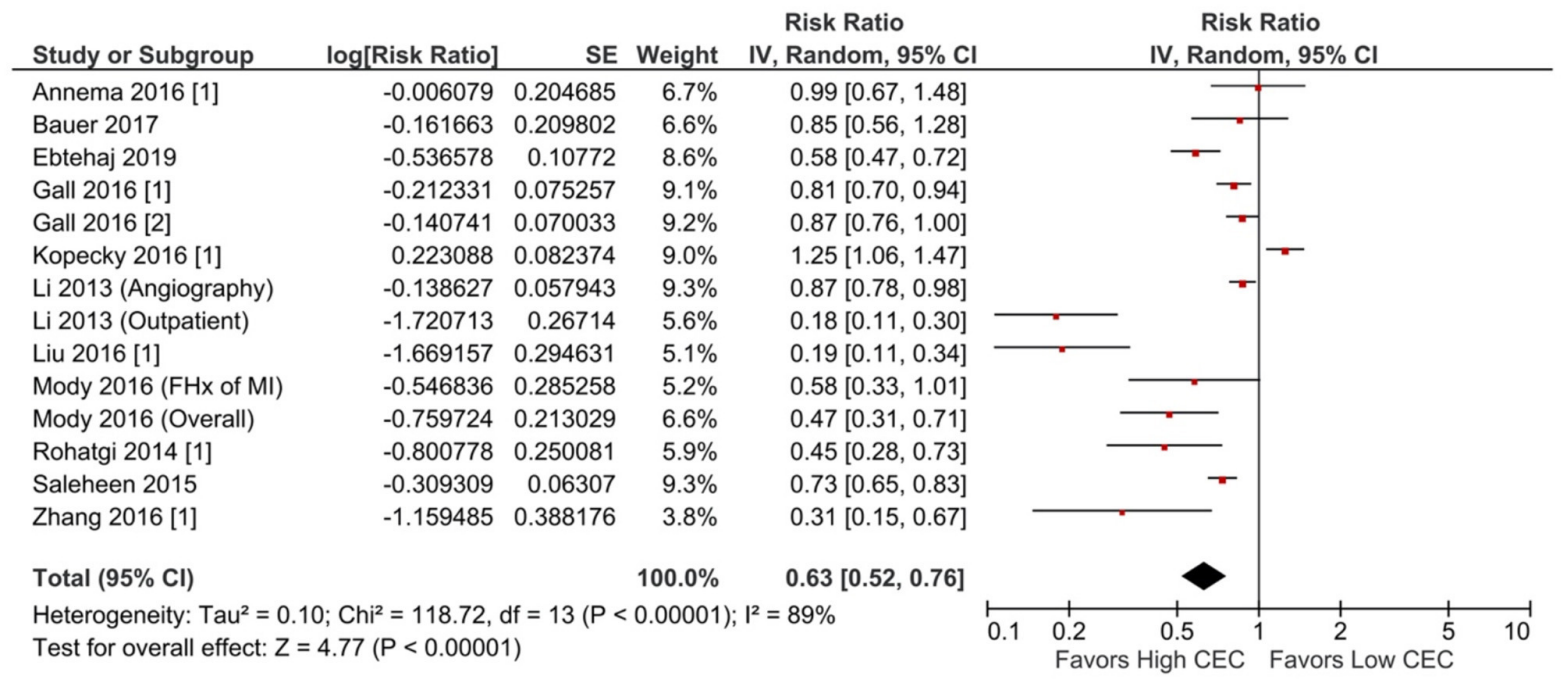
Adverse cardiovascular event: High CEC vs. low CEC (RR) (14 studies). CEC, cholesterol efflux capacity; CI, confidence interval; df, degree of freedom; FHx, family history; IV, inverse variance; MI, myocardial infarction; RR, risk ratio; SE, standard error.

**Figure 2:**
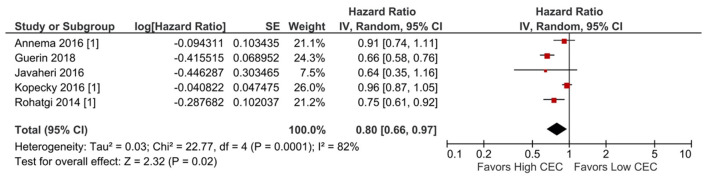
Adverse cardiovascular event: Per SD increment of CEC (HR) (5 studies). CEC, cholesterol efflux capacity; CI, confidence interval; df, degree of freedom; HR, hazard ratio; IV, inverse variance; SD, standard deviation; SE, standard error.

**Figure 3:**
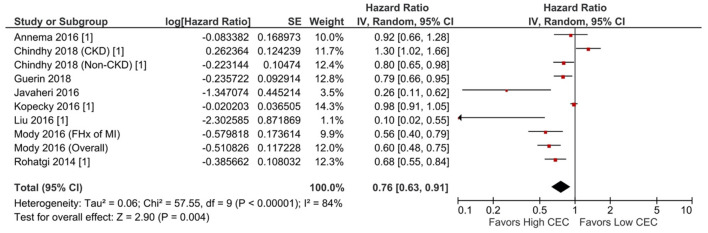
Adverse cardiovascular event: Per SD increment of CEC (adjusted HR) (10 studies). CEC, cholesterol efflux capacity; CI, confidence interval; CKD, chronic kidney disease; df, degree of freedom; FHx, family history; HR, hazard ratio; IV, inverse variance; MI, myocardial infarction; SE, standard error.

Increased CEC was significantly associated with reduced adverse cardiovascular events. Compared with the lowest CEC, the highest levels of CEC were associated with a 37% lower risk of adverse cardiovascular events (RR = 0.63; 95% CI, 0.52 to 0.76; *P* < 0.00001; Figure 1). Every SD increase of CEC was associated with a 20% lower risk of adverse cardiovascular events (HR = 0.80; 95% CI, 0.66–0.97; *P* = 0.02; Figure 2). The association remained significant even after adjusting for cardiovascular risk factors (e.g., with underlying or a history of CAD, dyslipidemia, family history of myocardial infarction), medications, and HDL-C levels (HR = 0.76; 95% CI, 0.63–0.91; *P* = 0.004; Figure 3). The I^2^ values ranged from 82 to 89%, indicating a high degree of heterogeneity. There were significant small-study effects as determined by the funnel plots (Supplementary Figures S16, S17) and Egger's test (Supplementary Table S6). After controlling for publication bias, high CEC remained associated with an improved cardiovascular outcome (RR = 0.79; 95% CI, 0.65–0.97; Supplementary Table S6), and the risk of adverse cardiovascular events was 19% lower with every SD increment of CEC (HR = 0.81; 95% CI, 0.66–0.98; Supplementary Table S6).

**Figure 4:**
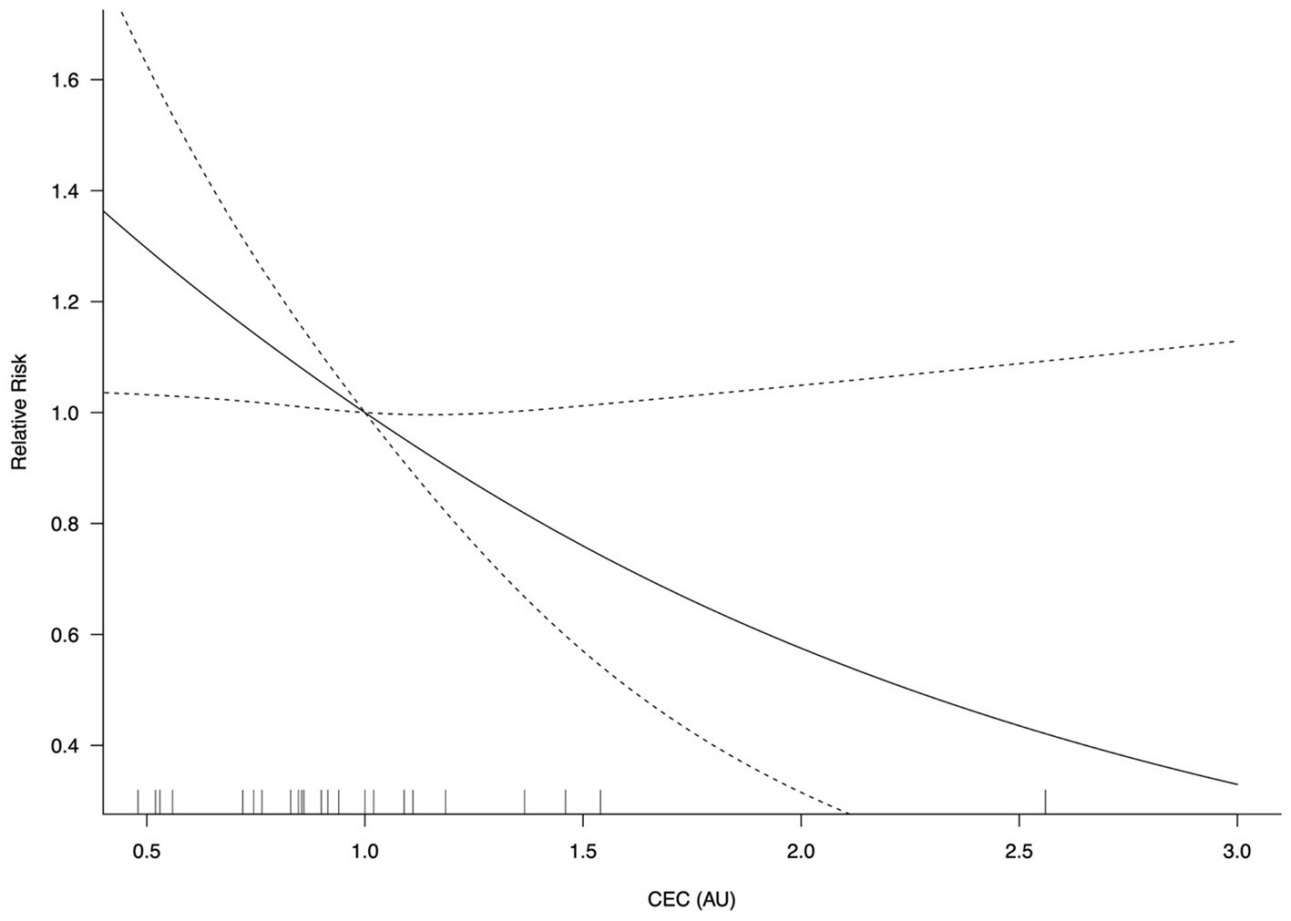
CEC-adverse cardiovascular event relative risk relationship (*P* = 0.024). AU, arbitrary unit; CEC, cholesterol efflux capacity; CI, confidence interval. Log-transformed CEC was used.

In the restricted cubic spline model, the relationship between CEC levels and adverse cardiovascular events are depicted in Figure 4. The risk of adverse cardiovascular events did not vary with CEC concentrations in a log-linear fashion (non-linearity *P* = 0.075). A significant CEC-adverse cardiovascular relative risk relationship was observed (*P* = 0.024) such that for every 0.1 unit increase in CEC, there was a 5% reduced risk for adverse cardiovascular events (RR = 0.95; 95% CI, 0.91–0.99).

Subgroup analyses on the association with adverse cardiovascular events are summarized in Supplementary Figures S19, S20. Compared with the low CEC group, the high CEC group had a lower risk among the individuals without cardiovascular risk factors or CKD (RR = 0.54; 95% CI, 0.41–0.71; *P* < 0.0001) and individuals with cardiovascular risk factors (RR = 0.54; 95% CI, 0.38–0.78; *P* = 0.001). Of note, the inverse relationship was not observed in CKD patients (RR = 1.08; 95% CI, 0.86–1.38; *P* = 0.50). The association with adverse cardiovascular events was heterogeneous across subgroups (*P* = 0.0002, I^2^ = 88.6%). Similarly, for each SD increment of CEC, a significantly decreased risk was observed in individuals without cardiovascular risk factors or CKD (HR = 0.69; 95% CI, 0.59 −0.82; *P* < 0.00001) and individuals with cardiovascular risk factors (HR = 0.49; 95% CI, 0.29–0.82; *P* = 0.006), but not in patients with CKD (HR = 1.05; 95% CI, 0.87–1.27; *P* = 0.62). There was a significant difference across subgroups (*P* = 0.0007, I^2^ = 86.3%).

### Association With Atherosclerotic Cardiovascular Disease

Higher CEC (i.e., better CEC) was significantly associated with lower ASCVD risk. Compared with the lowest CEC (i.e., worse CEC), the highest levels of CEC were associated with a 34% lower risk of ASCVD (RR = 0.66; 95% CI, 0.55–0.80; *P* < 0.0001; Supplementary Figure S2). After adjustment for cardiovascular risk factors, medications, and HDL-C levels, high CEC remained associated with a 21% lower risk compared with low CEC (RR = 0.79; 95% CI, 0.65–0.97; *P* = 0.02; Supplementary Figure S3). With respect to predicting ASCVD risk with each SD increase of CEC, there was no significant association either without adjustment (HR = 0.86; 95% CI, 0.68–1.10; *P* = 0.23; Supplementary Figure S4) or with adjustment (HR = 0.80; 95% CI, 0.64–1.00; *P* = 0.05; Supplementary Figure S5). With respect to differentiating ASCVD cases from controls, each SD increase of CEC was associated with a 20% lower odds of ASCVD (OR = 0.80; 95% CI, 0.66–0.97; *P* = 0.02; Supplementary Figure S6) and 19% lower odds after adjustment (OR = 0.81; 95% CI, 0.73–0.90; *P* = 0.0002; Supplementary Figure S7). The I^2^ values ranged from 79 to 91%, indicating a high degree of heterogeneity. There were significant small-study effects as determined by the funnel plots (Supplementary Figure S18) and Egger's test (Supplementary Table S6). After controlling for publication bias, high CEC remained associated with an improved cardiovascular outcome (RR = 0.78; 95% CI, 0.64–0.94; Supplementary Table S6).

Subgroup analysis on the association with ASCVD was summarized in Supplementary Figure S21. Compared with the low CEC group, the high CEC group had a lower risk among individuals without cardiovascular risk factors or CKD (RR = 0.54; 95% CI, 0.41–0.71; *P* < 0.0001) and individuals with cardiovascular risk factors (RR = 0.75; 95% CI, 0.60–0.93; *P* = 0.009). Of note, the inverse relationship was not observed in patients with CKD (RR = 1.08; 95% CI, 0.75–1.56; *P* = 0.67). There was a significant heterogeneity across subgroups (*P* = 0.01, I^2^ = 77.8%).

### Association With Death From All-Causes and Death From Cardiovascular Causes

The high CEC group did not have a significantly different risk of all-cause mortality compared with the low CEC group (RR = 0.61; 95% CI, 0.27–1.41; *P* = 0.25; Supplementary Figure S8). The risk of mortality did not vary significantly with per SD increment of CEC either without adjustment (HR = 0.81; 95% CI, 0.64–1.02; *P* = 0.08; Supplementary Figure S9) or with adjustment (HR = 0.77; 95% CI, 0.58 −1.02; *P* = 0.07; Supplementary Figure S10). The I^2^ values ranged from 81 to 94%, indicating a high degree of heterogeneity.

Similarly, a significant association between CEC and cardiovascular mortality was not observed. The high CEC group had a comparable risk of all-cause mortality with the low CEC group (RR = 0.48; 95% CI, 0.14–1.62; *P* = 0.24; Supplementary Figure S11). The risk of cardiovascular mortality did not vary per SD increment of CEC after adjustment (HR = 1.08; 95% CI, 0.72–1.62; *P* = 0.71; Supplementary Figure S12). The I^2^ values ranged from 71 to 89%, indicating a high degree of heterogeneity.

### Difference in CEC Between Cases and Controls

In this separate analysis of case-control studies, mean CEC levels between cases (individuals with adverse cardiovascular events, with ASCVD, or died) and controls (individuals without adverse cardiovascular events, without ASCVD, or survived) were compared. Compared with controls, a lower level of CEC was observed in cases who developed adverse cardiovascular events (mean difference, −0.08; 95% CI, −0.12 to −0.04; *P* < 0.00001; Supplementary Figure S13), cases who developed ASCVD (mean difference, −0.09; 95% CI, −0.16 to −0.02; *P* = 0.007; Supplementary Figure S14), and cases who died (mean difference, −0.07; 95% CI, −0.11 to −0.04; *P* < 0.0001; Supplementary Figure S15). The I^2^ values ranged from 83 to 92%, indicating a high degree of heterogeneity.

## Discussion

**Figure 5:**
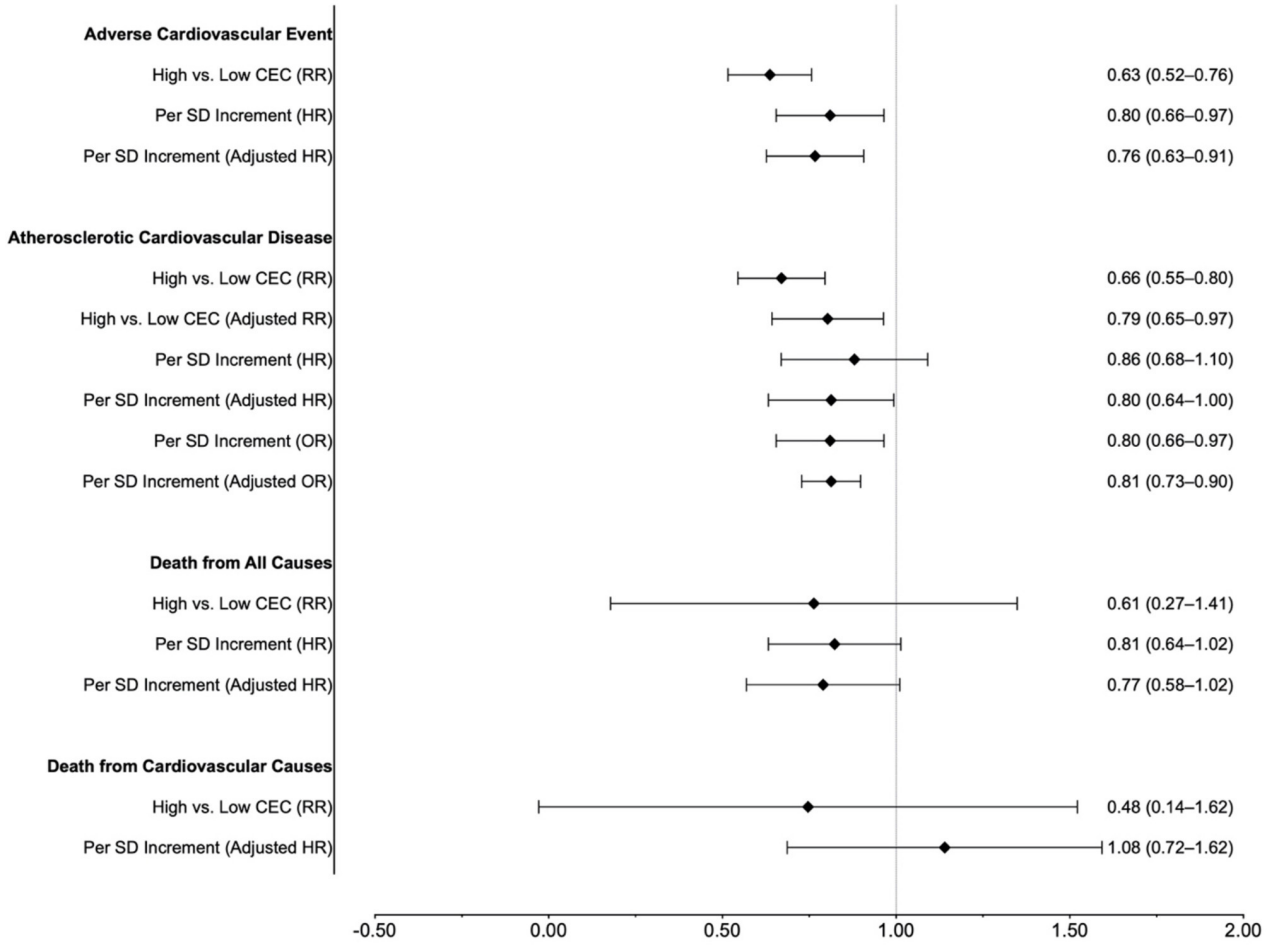
Summary of results.

In this meta-analysis, higher CEC levels were associated with favorable cardiovascular outcomes (Figure 5). Compared with the lowest CEC group, the highest CEC group had a 37 and 34% reduced risk of adverse cardiovascular events and ASCVD, respectively. Every SD increase in CEC [equivalent to 0.27 unit in the study by Ebtehaj et al. (10)] was associated with a 20% lower risk of adverse cardiovascular events. When fitting a restricted cubic spline model, there was an inverse concentration-dependent relationship, with a 5% lower risk of adverse cardiovascular events for every 0.1 unit increase of CEC.

The conventional “HDL hypothesis” posits that interventions that increase the plasma level of HDL-C reduce the risk of coronary heart disease. However, HDL-C-raising therapies, such as fibrates, niacin, and cholesteryl ester transfer protein inhibitors have not consistently demonstrated cardiovascular benefits (28). Furthermore, Mendelian randomization studies did not demonstrate a causal relationship between genetically-altered plasma HDL-C levels and cardiovascular risk (29–33). Rather than focusing on HDL-C levels, emerging evidence has highlighted the functional aspects of HDL in improving cardiovascular outcomes, known as the “HDL flux hypothesis”(34) In contrast to the HDL hypothesis, the HDL flux hypothesis postulates that interventions promoting CEC and reverse cholesterol transport may stabilize atherosclerosis and reduce the risk of coronary heart disease, regardless of whether it affects plasma HDL cholesterol levels (35). Similar to our findings, a previous meta-analysis of 14 studies showed that there was a relationship between CEC and cardiovascular risk (36). Additionally, the highest CEC group was associated with 44% reduced risk of cardiovascular events compared with the lowest CEC group, and per SD increase in CEC was associated 13% reduced risk.

Although the association of CEC with all-cause mortality was not statistically significant (Supplementary Figures S8–S10), the current analysis demonstrates that there may be a trend toward lower mortality with higher CEC. Among the three studies available for all-cause mortality sub-analysis (Supplementary Figure S8), the inverse association was evidence in one study where the adjusted HR of the highest CEC quartile compared to the lowest quartile was 0.24 (95% CI, 0.13–0.44; *P* < 0.001) (21). Moreover, deceased patients had significantly lower mean levels of CEC, as opposed to the survived patients (<0.0001; Supplementary Figure S15). Results from the current analysis of 20 studies were generally consistent with the work by Qiu et al. The present analysis further demonstrated that there was a significant difference in the mean CEC between cases (those who had adverse cardiovascular events or ASCVD) and controls. In addition to all-cause mortality, the present analysis shows that the association with cardiovascular mortality was not significant. Notably, the performance of CEC as a prognostic indicator of cardiovascular risk among patients with CKD was shown to be limited compared to its performance among patients with normal renal function. Of the three studies included in this analysis, the definitions of the CKD varied vastly, and included renal transplant recipients, (24) patients with eGFR 15 to 89 ml/min/1.73m^2^ (2, 16), and patients on hemodialysis (18). This suggests a significant heterogeneity in patients with CKD that were included in this analysis. Accordingly, further primary research among patients with CKD is required to explore whether CEC or other functional properties of HDL particles can assist with cardiovascular risk prediction.

The gold standard for measuring CEC in humans has not yet been established. It is possible that the choice of CEC assay may influence its association with adverse cardiovascular outcomes. For instance, the Cohort on Diabetes and Atherosclerosis Maastricht (CODAM) study found no association between CEC and subclinical or clinical atherosclerosis among participants with normal glucose metabolism, prediabetes, or diabetes using human THP-1 cells as the cholesterol donor (37). In contrast, a significant correlation was observed when remeasured using murine J774 cells among a subset of samples, suggesting the impact of cholesterol donor on CEC measurement (37). In this study, the majority (85%) of the included studies used J774 as the cholesterol donor. In the stratified analysis by the type of cholesterol donor (J774 vs. THP-1), high CEC was associated with a lower risk of adverse cardiovascular event among the 12 studies that used J774 as the donor (Supplementary Figure S22). In contrast, among the two studies that used THP-1 as the donor, high CEC was related to a greater risk. Regarding the association of per SD increment of CEC with adverse cardiovascular event (Supplementary Figures S23, S24), there was no significant difference between the J774 subgroup and THP-1 subgroup. More studies using THP-1 as the cholesterol donor are needed to examine the relationship between CEC and adverse cardiovascular event. In the stratified analysis by the cholesterol tracer ([^3^H]-Cholesterol vs. BODIPY-Cholesterol), high CEC was associated with a lower risk of adverse cardiovascular event among the 10 studies that used [^3^H]-Cholesterol as well as in the four studies that used BODIPY-Cholesterol (Supplementary Figure S25), with a greater magnitude of association observed in the BODIPY-Cholesterol subgroup (RR = 0.40 [95% CI, 0.26–0.61]) than in the [^3^H]-Cholesterol subgroup (RR = 0.74 [95% CI, 0.61–0.89]). Regarding the association of per SD increment of CEC with adverse cardiovascular event (Supplementary Figures S26, S27), there was no significant difference between the [^3^H]-Cholesterol subgroup and BODIPY-Cholesterol subgroup. Last, the difference in laboratory protocols (e.g., using whole serum vs. apolipoprotein B-depleted serum) across studies may have contributed to the heterogeneity of results.

Several interventions have been shown to improve the HDL function. For instance, in the STAMPEDE sub-study, bariatric surgeries, including Roux-en-Y gastric bypass and sleeve gastrectomy), were found to improve HDL functionality as evaluated by the CEC assay at five years. (38) In addition, eicosapentaenoic acid (EPA) supplementation has been associated with a dose-dependent increase of CEC from macrophages mediated by ATP-binding cassette transporter A1 (ABCA1) (39), which may help explain the anti-atherogenic properties and cardiovascular benefits of EPA in high-risk patients from recent trials (40). Furthermore, CETP inhibitors have been shown to significantly improve CEC along with HDL level (41–43). In further support of the HDL flux hypothesis, a novel infusible ApoA-I agent named CSL112 has been associated with an immediate and pronounced increase in CEC in patients with stable atherosclerotic disease and in healthy individuals (44). To test the safety and tolerability of CSL112, the AEGIS-I trial (ApoA-I Event Reducing in Ischemic Syndromes I) was a multicenter, randomized double blind placebo controlled trial that demonstrated four weekly infusions of CSL112 were feasible, well tolerated, and not associated with significant changes in hepatic or renal function among patients with an acute myocardial infarction (45). Importantly, the AEGIS-I trial demonstrated that compared with placebo, CSL112 was associated with improved CEC (45). To determine if improving cholesterol efflux is associated with improved cardiovascular outcomes, the AEGIS-II trial (ApoA-I Event Reducing in Ischemic Syndromes II) is underway and will evaluate the efficacy and safety of CSL112 in reducing the risk of major adverse cardiovascular events in patients with acute myocardial infarction.

### Limitations

This meta-analysis has several limitations that should be considered. First, the follow-up duration and case definitions for adverse cardiovascular events and ASCVD vary across the studies. Therefore, this analysis was unable to ascertain the association of CEC with specific components of the composite endpoints. Second, covariates included in the multivariable models (such as cardiovascular risk factors, medications, and the lipid panel) were not consistent and may impact the accuracy of the adjusted risk estimates. Third, only three endpoint comparisons had ten or fewer studies available. For this analysis, tests of small-study effect and subgroup analyses were performed. Few studies were available for examining the association with all-cause death and cardiovascular death; however, the lack of association with these endpoints may reflect a lack of statistical power. More data are warranted to validate the association of CEC with specific cardiovascular outcomes while accounting for individual risk profile and CEC method. Last, the cutoff value for defining high versus low CEC varied across the included studies. As this was a study-level meta-analysis based on aggregated data, a uniform cutoff value of CEC could not be applied to the analysis. Future patient-level meta-analysis is required to validate the findings.

## Conclusion

The meta-analysis demonstrates an inverse relationship between CEC levels, a quantitative measure of HDL functionality, and the risk of adverse cardiovascular events or atherosclerotic cardiovascular disease. Future studies should examine whether CEC can serve as a therapeutic target for improving cardiovascular outcomes.

## Data Availability Statement

The original contributions presented in the study are included in the article/Supplementary Material, further inquiries can be directed to the corresponding author.

## Author Contributions

JL and GC: concept and design, acquisition, analysis, or interpretation of data, and drafting of the manuscript. CF, SK, AK, SK, DD, AS, BK, RY, DB, and CG: critical revision of the manuscript for important intellectual content. CG: supervision. All authors have read and agreed to the published version of the manuscript.

## Funding

This study received funding from CSL Behring. The funder was not involved in the study design, collection, analysis, interpretation of data, the writing of this article or the decision to submit it for publication.

## Conflict of Interest

DD, AS, and BK are employed by CSL Behring. RY has received research grants from Abbott Vascular, AstraZeneca, Cook, BD Bard, Boston Scientific, Medtronic, and Philips; and is a consultant for Abbott Vascular, AstraZeneca, Boston Scientific, Edwards Lifesciences, Medtronic, Shockwave Medical, and Zoll. DB has served on advisory boards for Medscape Cardiology, the Boston VA Research Institute, the Society of Chest Pain Centers, and the American Heart Association Get With The Guidelines Science Subcommittee, received honoraria for consulting from the American College of Cardiology, the Duke Clinical Research Institute, Slack Publications, and WebMD, and has received research grants from Amarin, AstraZeneca, Bristol-Myers Squibb, Eisai, Ethicon, Medtronic, Sanofi Aventis, and The Medicines Company. CG institution receives funding from Johnson & Johnson and Bayer Healthcare, received grants from Abbott, Bayer, Genentech, Ikaria, Johnson & Johnson, Merck, and Sanofi-Aventis, received honoraria from Biogen IDEC, Bristol-Meyers Squibb, Daiichi Sankyo, CSL Behring, Cytori Therapeutics, Eli Lilly, GlaxoSmithkline, Genentech, Ischemix, Merck, Portola, Regado, Sanofi Aventis, The Medicines Company, and Medicure for consulting, and received compensation for lectures and service on speaking bureaus for Daiichi Sankyo, and Eli Lilly and Company. The remaining authors declare that the research was conducted in the absence of any commercial or financial relationships that could be construed as a potential conflict of interest.

## Publisher's Note

All claims expressed in this article are solely those of the authors and do not necessarily represent those of their affiliated organizations, or those of the publisher, the editors and the reviewers. Any product that may be evaluated in this article, or claim that may be made by its manufacturer, is not guaranteed or endorsed by the publisher.

## Abbreviations

ASCVD: atherosclerotic cardiovascular disease
AU: arbitrary unit
CAD: coronary artery disease
CEC: cholesterol efflux capacity
CI: confidence interval
CKD: chronic kidney disease
df: degree of freedom
eGFR: estimated glomerular filtration rate
HR: hazard ratio
HDL-C: high-density lipoprotein cholesterol
HR: hazard ratio
IV: inverse variance
OR: odds ratio
RR: relative risk
SD: standard deviation
SE: standard error.

## References

[1] Kappelle PJ Gansevoort RT Hillege JL Wolffenbuttel BH Dullaart RP. group Ps. apolipoprotein B/A-I and total cholesterol/high-density lipoprotein cholesterol ratios both predict cardiovascular events in the general population independently of nonlipid risk factors, albuminuria and C-reactive protein. J Intern Med. (2011) 269:232–42. 10.1111/j.1365-2796.2010.02323.x 21129046

[2] Nicholls SJ Tuzcu EM Sipahi I Grasso AW Schoenhagen P Hu T . Statins, high-density lipoprotein cholesterol, and regression of coronary atherosclerosis. JAMA. (2007) 297:499–508. 10.1001/jama.297.5.499 17284700

[3] Haynes R Valdes-Marquez E Hopewell JC Chen F Li J Parish S . Members HTWC, members HTSC. Serious adverse effects of extended-release niacin/laropiprant: results from the heart protection study 2-treatment of HDL to reduce the incidence of vascular events (HPS2-THRIVE). Trial Clin Ther. (2019) 41:1767–77. 10.1016/j.clinthera.2019.06.012 31447131

[4] Nicholls SJ. The AIM-HIGH (Atherothrombosis Intervention in Metabolic Syndrome With Low HDL/High Triglycerides: Impact on Global Health Outcomes) trial: to believe or not to believe? J Am Coll Cardiol. (2012) 59:2065–7. 10.1016/j.jacc.2012.02.021 22520248

[5] Burgess S Harshfield E. Mendelian randomization to assess causal effects of blood lipids on coronary heart disease: lessons from the past and applications to the future. Curr Opin Endocrinol Diabetes Obes. (2016) 23:124–30. 10.1097/MED.0000000000000230 26910273 PMC4816855

[6] Musunuru K Kathiresan S. Surprises from genetic analyses of lipid risk factors for atherosclerosis. Circ Res. (2016) 118:579–85. 10.1161/CIRCRESAHA.115.306398 26892959 PMC4762058

[7] Haase CL Tybjaerg-Hansen A Qayyum AA Schou J Nordestgaard BG Frikke-Schmidt R . HDL cholesterol and ischemic cardiovascular disease: a Mendelian randomization study of HDL cholesterol in 54,500 individuals. J Clin Endocrinol Metab. (2012) 97:E248–256. 10.1210/jc.2011-1846 22090275

[8] Rohatgi A Khera A Berry JD Givens EG Ayers CR Wedin KE . cholesterol efflux capacity and incident cardiovascular events. N Engl J Med. (2014) 371:2383–93. 10.1056/NEJMoa1409065 25404125 PMC4308988

[9] Saleheen D Scott R Javad S Zhao W Rodrigues A Picataggi A . Association of HDL cholesterol efflux capacity with incident coronary heart disease events: a prospective case-control study. Lancet Diabetes Endocrinol. (2015) 3:507–13. 10.1016/S2213-8587(15)00126-6 26025389 PMC4648056

[10] Ebtehaj S Gruppen EG Bakker SJL Dullaart RPF Tietge UJF. HDL (High-density lipoprotein) cholesterol efflux capacity is associated with incident cardiovascular disease in the general population arterioscler. Thromb Vasc Biol. (2019) 39:1874–83. 10.1161/ATVBAHA.119.312645 31315436

[11] Cahill LE Sacks FM Rimm EB Jensen MK. Cholesterol efflux capacity, HDL cholesterol, and risk of coronary heart disease: a nested case-control study in men. J Lipid Res. (2019) 60:1457–64. 10.1194/jlr.P093823 31142574 PMC6672045

[12] Guerin M Silvain J Gall J Darabi M Berthet M Frisdal E . Association of serum cholesterol efflux capacity with mortality in patients with ST-segment elevation myocardial infarction. J Am Coll Cardiol. (2018) 72:3259–69. 10.1016/j.jacc.2018.09.080 30573028

[13] Chindhy S Joshi P Khera A Ayers CR Hedayati SS Rohatgi A. Impaired renal function on cholesterol efflux capacity, HDL particle number, and cardiovascular events. J Am Coll Cardiol. (2018) 72:698–700. 10.1016/j.jacc.2018.05.043 30072004 PMC6152844

[14] Tejera-Segura B Macia-Diaz M Machado JD de Vera-Gonzalez A Garcia-Dopico JA Olmos JM . Cholesterol efflux capacity in rheumatoid arthritis patients: contributing factors and relationship with subclinical atherosclerosis. Arthritis Res Ther. (2017) 19:113. 10.1186/s13075-017-1311-3 28569219 PMC5452399

[15] Khera AV Demler OV Adelman SJ Collins HL Glynn RJ Ridker PM . Cholesterol efflux capacity, high-density lipoprotein particle number, and incident cardiovascular events: an analysis from the JUPITER trial (justification for the use of statins in prevention: an intervention trial evaluating rosuvastatin). Circulation. (2017) 135:2494–504. 10.1161/CIRCULATIONAHA.116.025678 28450350 PMC5490983

[16] Bauer L Kern S Rogacev KS Emrich IE Zawada A Fliser D . Cholesterol efflux capacity and cardiovascular events in patients with chronic kidney disease. J Am Coll Cardiol. (2017) 69:246–7. 10.1016/j.jacc.2016.10.054 28081833

[17] Gall J Frisdal E Bittar R Le Goff W Bruckert E Lesnik P . Association of cholesterol efflux capacity with clinical features of metabolic syndrome: relevance to atherosclerosis. J Am Heart Assoc. (2016) 5:e004808. 10.1161/JAHA.116.004808 27881422 PMC5210394

[18] Kopecky C Ebtehaj S Genser B Drechsler C Krane V Antlanger M . Cholesterol efflux does not predict cardiovascular risk in hemodialysis patients. J Am Soc Nephrol. (2017) 28:769–75. 10.1681/ASN.2016030262 27612996 PMC5328158

[19] Javaheri A Molina M Zamani P Rodrigues A Novak E Chambers S . Cholesterol efflux capacity of high-density lipoprotein correlates with survival and allograft vasculopathy in cardiac transplant recipients. J Heart Lung Transplant. (2016) 35:1295–302. 10.1016/j.healun.2016.06.022 27498384 PMC5107129

[20] Mody P Joshi PH Khera A Ayers CR Rohatgi A. Beyond coronary calcification, family history, and c-reactive protein: cholesterol efflux capacity and cardiovascular risk prediction. J Am Coll Cardiol. (2016) 67:2480–7. 10.1016/j.jacc.2016.03.538 27230043 PMC4884307

[21] Liu C Zhang Y Ding D Li X Yang Y Li Q . Cholesterol efflux capacity is an independent predictor of all-cause and cardiovascular mortality in patients with coronary artery disease: A prospective cohort study. Atherosclerosis. (2016) 249:116–24. 10.1016/j.atherosclerosis.2015.10.111 27088866

[22] Zhang J Xu J Wang J Wu C Xu Y Wang Y . Prognostic usefulness of serum cholesterol efflux capacity in patients with coronary artery disease. Am J Cardiol. (2016) 117:508–14. 10.1016/j.amjcard.2015.11.033 26718234

[23] Ogura M Hori M Harada-Shiba M. Association between cholesterol efflux capacity and atherosclerotic cardiovascular disease in patients with familial hypercholesterolemia. Arterioscler Thromb Vasc Biol. (2016) 36:181–8. 10.1161/ATVBAHA.115.306665 26543100

[24] Annema W Dikkers A de Boer JF Dullaart RP Sanders JS Bakker SJ . Cholesterol efflux predicts graft failure in renal transplant recipients. J Am Soc Nephrol. (2016) 27:595–603. 10.1681/ASN.2014090857 26319244 PMC4731105

[25] Ishikawa T Ayaori M Uto-Kondo H Nakajima T Mutoh M Ikewaki K. High-density lipoprotein cholesterol efflux capacity as a relevant predictor of atherosclerotic coronary disease. Atherosclerosis. (2015) 242:318–22. 10.1016/j.atherosclerosis.2015.06.028 26246268

[26] Li XM Tang WH Mosior MK Huang Y Wu Y Matter W . Paradoxical association of enhanced cholesterol efflux with increased incident cardiovascular risks. Arterioscler Thromb Vasc Biol. (2013) 33:1696–705. 10.1161/ATVBAHA.113.301373 23520163 PMC3743250

[27] Khera AV Cuchel M de la Llera-Moya M Rodrigues A Burke MF Jafri K . Cholesterol efflux capacity, high-density lipoprotein function, and atherosclerosis. N Engl J Med. (2011) 364:127–35. 10.1056/NEJMoa1001689 21226578 PMC3030449

[28] Xiang AS Kingwell BA. Rethinking good cholesterol: a clinicians' guide to understanding HDL. Lancet Diabetes Endocrinol. (2019) 7:575–82. 10.1016/S2213-8587(19)30003-8 30910502

[29] Frikke-Schmidt R Nordestgaard BG Stene MC Sethi AA Remaley AT Schnohr P . Association of loss-of-function mutations in the ABCA1 gene with high-density lipoprotein cholesterol levels and risk of ischemic heart disease. JAMA. (2008) 299:2524–32. 10.1001/jama.299.21.2524 18523221

[30] Johannsen TH Kamstrup PR Andersen RV Jensen GB Sillesen H Tybjaerg-Hansen A . Hepatic lipase, genetically elevated high-density lipoprotein, and risk of ischemic cardiovascular disease. J Clin Endocrinol Metab. (2009) 94:1264–73. 10.1210/jc.2008-1342 19088157

[31] Haase CL Tybjaerg-Hansen A Grande P Frikke-Schmidt R. Genetically elevated apolipoprotein A-I, high-density lipoprotein cholesterol levels, and risk of ischemic heart disease. J Clin Endocrinol Metab. (2010) 95:E500–510. 10.1210/jc.2010-0450 20826588

[32] Voight BF Peloso GM Orho-Melander M Frikke-Schmidt R Barbalic M Jensen MK . Plasma HDL cholesterol and risk of myocardial infarction: a mendelian randomisation study. Lancet. (2012) 380:572–80. 10.1016/S0140-6736(12)60312-2 22607825 PMC3419820

[33] Holmes MV Asselbergs FW Palmer TM Drenos F Lanktree MB Nelson CP . Mendelian randomization of blood lipids for coronary heart disease. Eur Heart J. (2015) 36:539–50. 10.1093/eurheartj/eht571 24474739 PMC4344957

[34] Rader DJ Tall AR. The not-so-simple HDL story: Is it time to revise the HDL cholesterol hypothesis? Nat Med. (2012) 18:1344–6. 10.1038/nm.2937 22961164

[35] Hewing B Parathath S Barrett T Chung WK Astudillo YM Hamada T . Effects of native and myeloperoxidase-modified apolipoprotein a-I on reverse cholesterol transport and atherosclerosis in mice. Arterioscler Thromb Vasc Biol. (2014) 34:779–89. 10.1161/ATVBAHA.113.303044 24407029 PMC3966977

[36] Qiu C Zhao X Zhou Q Zhang Z. High-density lipoprotein cholesterol efflux capacity is inversely associated with cardiovascular risk: a systematic review and meta-analysis. Lipids Health Dis. (2017) 16:212. 10.1186/s12944-017-0604-5 29126414 PMC5681808

[37] Josefs T Wouters K Tietge UJF Annema W Dullaart RPF Vaisar T . High-density lipoprotein cholesterol efflux capacity is not associated with atherosclerosis and prevalence of cardiovascular outcome: The CODAM study. J Clin Lipidol. (2020) 14:122–32 e124. 10.1016/j.jacl.2019.10.012 31791716 PMC8176544

[38] Lorkowski SW Brubaker G Rotroff DM Kashyap SR Bhatt DL Nissen SE . Bariatric surgery improves hdl function examined by apoa1 exchange rate and cholesterol efflux capacity in patients with obesity and type 2 diabetes. Biomolecules. (2020) 10:551. 10.3390/biom10040551 32260470 PMC7226587

[39] Dakroub H Nowak M Benoist J-F Paul J-L Fournier N. Eicosapentaenoic acid (EPA) increases ABCA1-medited cholesterol efflux from THP-1 human macrophages. Biochim Biophys Acta Mol Cell Biol Lipids. 1866:159016. 10.1016/j.bbalip.2021.159016 34332075

[40] Lakshmanan S Shekar C Kinninger A Dahal S Onuegbu A Cai AN . Association of high-density lipoprotein levels with baseline coronary plaque volumes by coronary CTA in the EVAPORATE trial. Atherosclerosis. (2020) 305:34–41. 10.1016/j.atherosclerosis.2020.05.014 32615321

[41] Nicholls SJ Ruotolo G Brewer HB Kane JP Wang MD Krueger KA . Cholesterol efflux capacity and pre-beta-1 HDL concentrations are increased in dyslipidemic patients treated with evacetrapib. J Am Coll Cardiol. (2015) 66:2201–10. 10.1016/j.jacc.2015.09.013 26564598

[42] van Capelleveen JC Kastelein JJ Zwinderman AH van Deventer SJ Collins HL Adelman SJ . Effects of the cholesteryl ester transfer protein inhibitor, TA-8995, on cholesterol efflux capacity and high-density lipoprotein particle subclasses. J Clin Lipidol. (2016) 10:1137–44. 10.1016/j.jacl.2016.06.006 27678430

[43] Metzinger MP Saldanha S Gulati J Patel KV El-Ghazali A Deodhar S . Effect of anacetrapib on cholesterol efflux capacity: a substudy of the DEFINE trial. J Am Heart Assoc. (2020) 9:e018136. 10.1161/JAHA.120.018136 33263263 PMC7955402

[44] Gille A D'Andrea D Tortorici MA Hartel G Wright SD. CSL112 (Apolipoprotein A-I [Human]) enhances cholesterol efflux similarly in healthy individuals and stable atherosclerotic disease patients. Arterioscler Thromb Vasc Biol. (2018) 38:953–63. 10.1161/ATVBAHA.118.310538 29437574 PMC5895137

[45] Michael Gibson C Korjian S Tricoci P Daaboul Y Yee M Jain P . Safety and tolerability of CSL112, a reconstituted, infusible, plasma-derived apolipoprotein A-I, after acute myocardial infarction: The AEGIS-I trial (ApoA-I event reducing in ischemic syndromes I). Circulation. (2016) 134:1918–30. 10.1161/CIRCULATIONAHA.116.025687 27881559 PMC5147036

