# Supplementary material for: Cholesterol Efflux Capacity and Its Association With Adverse Cardiovascular Events: A Systematic Review and Meta-Analysis
Source: Front Cardiovasc Med. 2021 Dec 13;8:774418. doi: 10.3389/fcvm.2021.774418 (PMC8710716; doi:10.3389/fcvm.2021.774418)

Supplemental Tables and Figures

Table S1. Detailed Queries
Table S2. Quality Assessment Criteria for Case-Control Studies
Table S3. Quality Assessment Form for Case-Control Studies
Table S4. Quality Assessment Criteria for Cohort Studies
Table S5. Quality Assessment Form for Cohort Studies
Table S6. Summary of Egger's Test for Small-Study Effect and Trim-and-Fill Method for Publication Bias
Figure S1. PRISMA Flow Diagram
Figure S2. Atherosclerotic Cardiovascular Disease: High CEC vs. Low CEC (RR) (12 studies)
Figure S3. Atherosclerotic Cardiovascular Disease: High CEC vs. Low CEC (Adjusted RR) (7 studies)
Figure S4. Atherosclerotic Cardiovascular Disease: Per SD Increment of CEC (HR) (2 studies)
Figure S5. Atherosclerotic Cardiovascular Disease: Per SD Increment of CEC (Adjusted HR) (6 studies)
Figure S6. Atherosclerotic Cardiovascular Disease: Per SD Increment of CEC (OR) (5 studies)
Figure S7. Atherosclerotic Cardiovascular Disease: Per SD Increment of CEC (Adjusted OR) (8 studies)
Figure S8. Death from All Causes: High CEC vs. Low CEC (RR) (3 studies)
Figure S9. Death from All Causes: Per SD Increment of CEC (HR) (4 studies)
Figure S10. Death from All Causes: Per SD Increment of CEC (Adjusted HR) (5 studies)
Figure S11. Death from Cardiovascular Causes: High CEC vs. Low CEC (RR) (4 studies)
Figure S12. Death from Cardiovascular Causes: Per SD Increment of CEC (Adjusted HR) (4 studies)
Figure S13. Adverse Cardiovascular Event: Mean Difference in CEC (6 studies)
Figure S14. Atherosclerotic Cardiovascular Disease: Mean Difference in CEC (4 studies)
Figure S15. Death from All Causes: Mean Difference in CEC (2 studies)
Figure S16. Funnel Plot of Adverse Cardiovascular Event: High CEC vs. Low CEC (RR) (14 studies)
Figure S17. Funnel Plot of Adverse Cardiovascular Event: Per SD Increment of CEC (Adjusted HR) (10 studies)
Figure S18. Funnel Plot of Atherosclerotic Cardiovascular Disease: High CEC vs. Low CEC (RR) (12 studies)
Figure S19. Subgroup Analysis of Adverse Cardiovascular Event: High CEC vs. Low CEC (RR) (14 studies)
Figure S20. Subgroup Analysis of Adverse Cardiovascular Event: Per SD Increment of CEC (Adjusted HR) (10 studies)
Figure S21. Subgroup Analysis of Atherosclerotic Cardiovascular Disease: High CEC vs. Low CEC (RR) (12 studies)
Figure S22. Adverse Cardiovascular Event Stratified by the Type of Cholesterol Donor: High CEC vs. Low CEC (RR) (14 studies)
Figure S23. Adverse Cardiovascular Event Stratified by the Type of Cholesterol Donor: Per SD Increment of CEC (HR) (5 studies)
Figure S24. Adverse Cardiovascular Event Stratified by the Type of Cholesterol Donor: Per SD Increment of CEC (adjusted HR) (10 studies)
Figure S25. Adverse Cardiovascular Event Stratified by the Method of Labeling: High CEC vs. Low CEC (RR) (14 studies)
Figure S26. Adverse Cardiovascular Event Stratified by the Method of Labeling: Per SD Increment of CEC (HR) (5 studies)
Figure S27. Adverse Cardiovascular Event Stratified by the Method of Labeling: Per SD Increment of CEC (adjusted HR) (10 studies)

Table S1. Detailed Queries

Database	Queries
Embase	('mortality'/exp OR 'death'/exp OR 'cardiovascular disease'/exp OR 'coronary heart disease'/exp OR 'acute coronary syndrome'/exp OR 'myocardial infarction'/exp OR 'cerebrovascular event') AND 'cholesterol efflux capacity':ab AND ([article]/lim OR [article in press]/lim OR [conference abstract]/lim OR [conference paper]/lim) AND [english]/lim AND ([embase]/lim OR [medline]/lim OR [embase classic]/lim OR [pubmed-not-medline]/lim)
PubMed	cholesterol efflux capacity[tw] AND (mortality[tw] OR death[tw] OR cardiovascular disease[tw] OR coronary heart disease[tw] OR acute coronary syndrome[tw] OR myocardial infarction[tw] OR stroke[tw] OR cerebrovascular event[tw]) AND Journal Article[pt]
Web of Science	(((TS =(cholesterol efflux capacity)) AND ((TS =(mortality OR death OR cardiovascular disease OR coronary heart disease OR acute coronary syndrome OR myocardial infarction OR stroke OR cerebrovascular event))))) AND LANGUAGE: (English) AND DOCUMENT TYPES: (Article OR Abstract of Published Item)

Table S2. Quality Assessment Criteria for Case-Control Studies

Domain	Item	Criterion	Rating
Selection	Is the Case Definition Adequate? (0 to 2)	Requires some independent validation (e.g. >1 person/record/time/process to extract information, or reference to primary record source such as x-rays or medical/hospital records).	2
		Record linkage (e.g. ICD codes in database) or self-report with no reference to primary record.	1
		No description.	0
	Representativeness of the Cases (0 to 1)	All eligible cases with outcome of interest over a defined period of time, all cases in a defined catchment area, all cases in a defined hospital or clinic, group of hospitals, health maintenance organization, or an appropriate sample of those cases (e.g. random sample).	1
		Not satisfying abovementioned requirements with potential for selection biases, or not stated.	0
	Selection of Controls (0 to 1)	Community controls (i.e. same community as cases and would be cases if had outcome).	1
		Hospital controls, within same community as cases (i.e. not another city) but derived from a hospitalized population.	1
		No description.	0
	Definition of Controls (0 to 1)	If cases are first occurrence of outcome, then it must explicitly state that controls have no history of this outcome. If cases have new (not necessarily first) occurrence of outcome, then controls with previous occurrences of outcome of interest should not be excluded.	1
		No mention of history of outcome.	0
Comparability	Matched or Adjusted by Key Factors (0 to 1)	Matched or adjusted by key factors including age, sex, and cardiovascular risk factors.	1
	Matched or Adjusted by Additional Factors (0 to 1)	Matched or adjusted by additional factors including total cholesterol, high-density lipoprotein cholesterol, low-density lipoprotein cholesterol, or triglycerides.	1
Exposure	Ascertainment of Exposure (0 to 1)	Secure records (e.g. medical records/research records)	1
		Self-reported records only, or not stated.	0
	Non-Response Rate (0 to 1)	Non-response rate is described.	1
		No description.	0

Table S3. Quality Assessment Form for Case-Control Studies

Study	Selection				Comparability		Outcome		Total Score
	Is the Case Definition Adequate? (0 to 2)	Representativeness of the Cases (0 to 1)	Selection of Controls (0 to 1)	Definition of Controls (0 to 1)	Matched or Adjusted by Key Factors (0 to 1)	Matched or Adjusted by Additional Factors (0 to 1)	Ascertainment of Exposure (0 to 1)	Non-Response Rate (0 to 1)	
Ebtehaj 2019	1	1	1	1	1	1	1	1	8
Cahill 2019	2	0	1	1	1	1	1	1	8
Tejera-Segura 2017	1	1	0	1	1	1	1	0	6
Khera 2017	1	1	1	1	1	1	1	1	8
Gall 2016	1	1	1	1	1	1	1	1	8
Ishikawa 2015	2	1	1	1	1	1	1	0	8
Saleheen 2015	1	1	1	1	1	1	1	0	7
Li 2013	1	1	1	1	1	1	1	0	7

Table S4. Quality Assessment Criteria for Cohort Studies

Domain	Item	Criterion	Rating
Selection	Representativeness of the Exposed Cohort (0 to 1)	Exposed individuals are representative in the community.	1
		No description.	0
	Selection of the Non-Exposed Cohort (0 to 1)	Drawn from the same community as the exposed cohort.	1
		No description.	0
	Ascertainment of Exposure (0 to 1)	Secure records (e.g. medical records/research records).	1
		No description.	0
	Demonstration That Outcome of Interest Was Not Present at Start of Study (0 to 1)	Yes	1
		No description.	0
Comparability	Matched or Adjusted by Key Factors (0 to 1)	Matched or adjusted by key factors, including age, sex, and cardiovascular risk factors.	1
	Matched or Adjusted by Additional Factors (0 to 1)	Matched or adjusted by additional factors including total cholesterol, high-density lipoprotein cholesterol, low-density lipoprotein cholesterol, or triglycerides.	1
Outcome	Assessment of Outcome (0 to 1)	Independent or blind assessment, confirmation of the outcome by reference to secure records (e.g. medical records/research records), or record linkage (e.g. identified through ICD codes on database records).	1
		Self-reported records only, or not stated.	0
	Was Follow-Up Long Enough for Outcomes to Occur (0 to 1)	Yes (at least one year).	1
		Less than one year or no description.	0
	Adequacy of Follow Up of Cohorts (0 to 1)	At least 80% of subjects complete the follow-up, and subjects lost to follow-up are unlikely to introduce bias.	1
		Follow-up rate < 80% and no description of subjects lost to follow-up.	0

Table S5. Quality Assessment Form for Cohort Studies

Study	Selection				Comparability		Outcome			Total Score
	Representativeness of the Exposed Cohort (0 to 1)	Selection of the Non-Exposed Cohort (0 to 1)	Ascertainment of Exposure (0 to 1)	Demonstration That Outcome of Interest Was Not Present at Start of Study (0 to 1)	Matched or Adjusted by Key Factors (0 to 1)	Matched or Adjusted by Additional Factors (0 to 1)	Assessment of Outcome (0 to 1)	Was Follow-Up Long Enough for Outcomes to Occur (0 to 1)	Adequacy of Follow Up of Cohorts (0 to 1)	
Guerin 2018	0	1	1	1	1	1	1	1	0	7
Chindhy 2018	1	1	1	1	1	1	1	1	0	8
Bauer 2017	1	1	1	1	1	1	0	1	0	7
Kopecky 2016	1	1	1	0	1	1	1	1	0	7
Javaheri 2016	1	1	1	0	1	1	1	1	0	7
Mody 2016	1	1	1	1	1	1	1	1	1	9
Liu 2016	1	1	1	1	1	1	1	1	1	9
Zhang 2016	0	1	1	1	1	1	1	1	1	8
Ogura 2016	0	1	1	0	1	1	1	0	0	5
Annema 2016	0	1	1	1	1	1	1	1	1	8
Rohatgi 2014	1	1	1	1	1	1	1	1	1	9
Khera 2011	1	1	1	1	1	1	1	0	0	7

Table S6. Summary of Egger's Test for Small-Study Effect and Trim-and-Fill Method for Publication Bias

Endpoint	Egger's Test for Small-Study Effects				Effect of Observed Studies				Effect of Observed plus Imputed Studies			
	β1	SE	z	P-value	Number of Studies	Point Estimate	95% LCL	95% UCL	Number of Studies	Point Estimate	95% LCL	95% UCL
Adverse Cardiovascular Event: High CEC vs. Low CEC (RR)	-3.84	0.852	-4.51	<0.0001	14	0.631	0.522	0.762	18	0.790	0.646	0.966
Adverse Cardiovascular Event: Per SD Increment of CEC (adjusted HR)	-2.79	0.837	-3.33	0.0009	10	0.758	0.628	0.914	12	0.807	0.664	0.980
Atherosclerotic Cardiovascular Disease: High CEC vs. Low CEC (RR)	-3.58	0.870	-4.11	<0.0001	12	0.662	0.550	0.797	15	0.775	0.638	0.941

Abbreviations: CEC, cholesterol efflux capacity; HR, hazard ratio; LCL, lower confidence limit; RR, risk ratio; SD, standard deviation; SE, standard error; UCL, upper confidence limit.

Figure S1. PRISMA Flow Diagram

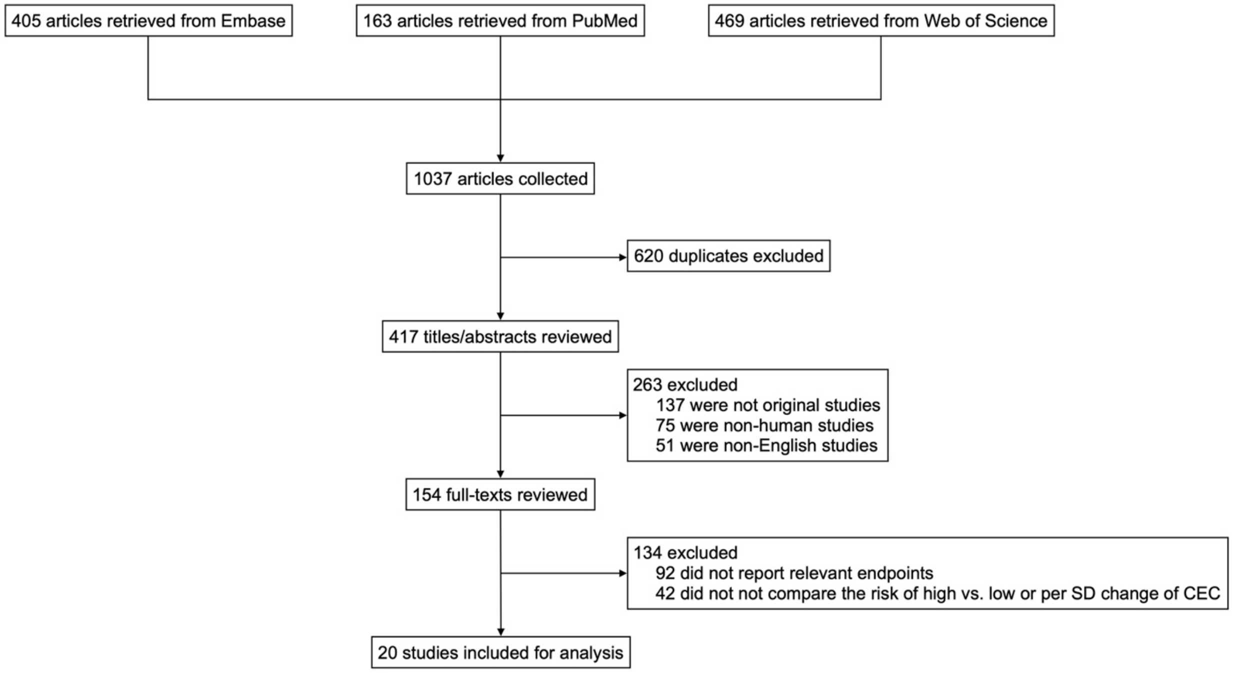

Figure S2. Atherosclerotic Cardiovascular Disease: High CEC vs. Low CEC (RR) (12 studies)

Study or Subgroup	log[Risk Ratio]	SE	Weight	Risk Ratio IV, Random, 95% CI
Bauer 2017	-0.161663	0.209802	7.3%	0.85 [0.56, 1.28]
Ebtehaj 2019	-0.536578	0.10772	9.9%	0.58 [0.47, 0.72]
Gall 2016 [1]	-0.212331	0.075257	10.6%	0.81 [0.70, 0.94]
Gall 2016 [2]	-0.140741	0.070033	10.7%	0.87 [0.76, 1.00]
Kopecky 2016 [1]	0.223088	0.082374	10.5%	1.25 [1.06, 1.47]
Li 2013 (Angiography)	-0.138627	0.057943	10.9%	0.87 [0.78, 0.98]
Li 2013 (Outpatient)	-1.720713	0.26714	6.0%	0.18 [0.11, 0.30]
Mody 2016 (FHx of MI)	-0.546836	0.285258	5.6%	0.58 [0.33, 1.01]
Mody 2016 (Overall)	-0.759724	0.213029	7.2%	0.47 [0.31, 0.71]
Rohatgi 2014 [1]	-0.800778	0.250081	6.3%	0.45 [0.28, 0.73]
Saleheen 2015	-0.309309	0.06307	10.9%	0.73 [0.65, 0.83]
Zhang 2016 [1]	-1.159485	0.388176	3.9%	0.31 [0.15, 0.67]
**Total (95% CI)**			**100.0%**	**0.66 [0.55, 0.80]**

Heterogeneity: Tau² = 0.08; Chi² = 93.43, df = 11 (P < 0.00001); I² = 88%

Test for overall effect: Z = 4.37 (P < 0.0001)

Risk Ratio IV, Random, 95% CI

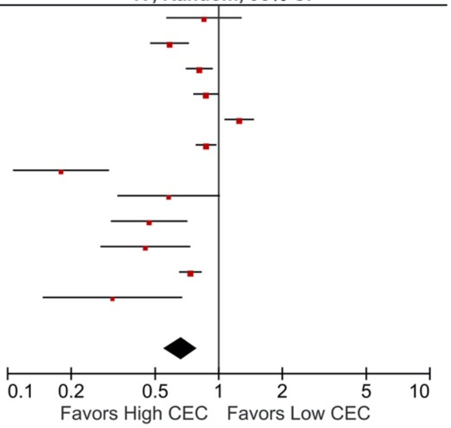

Figure S3. Atherosclerotic Cardiovascular Disease: High CEC vs. Low CEC (Adjusted RR) (7 studies)

Study or Subgroup	log[Risk Ratio]	SE	Weight	Risk Ratio IV, Random, 95% CI
Cahill 2019	0.425268	0.362833	5.7%	1.53 [0.75, 3.12]
Gall 2016 [1]	-0.228822	0.093972	17.2%	0.80 [0.66, 0.96]
Gall 2016 [2]	-0.099265	0.076994	18.1%	0.91 [0.78, 1.05]
Khera 2011	-0.39171	0.15096	14.0%	0.68 [0.50, 0.91]
Li 2013 (Angiography)	0.049738	0.049623	19.2%	1.05 [0.95, 1.16]
Li 2013 (Outpatient)	-1.431373	0.294691	7.5%	0.24 [0.13, 0.43]
Saleheen 2015	-0.248087	0.070094	18.4%	0.78 [0.68, 0.90]
**Total (95% CI)**			**100.0%**	**0.79 [0.65, 0.97]**

Heterogeneity: Tau² = 0.05; Chi² = 41.62, df = 6 (P < 0.00001); I² = 86%

Test for overall effect: Z = 2.27 (P = 0.02)

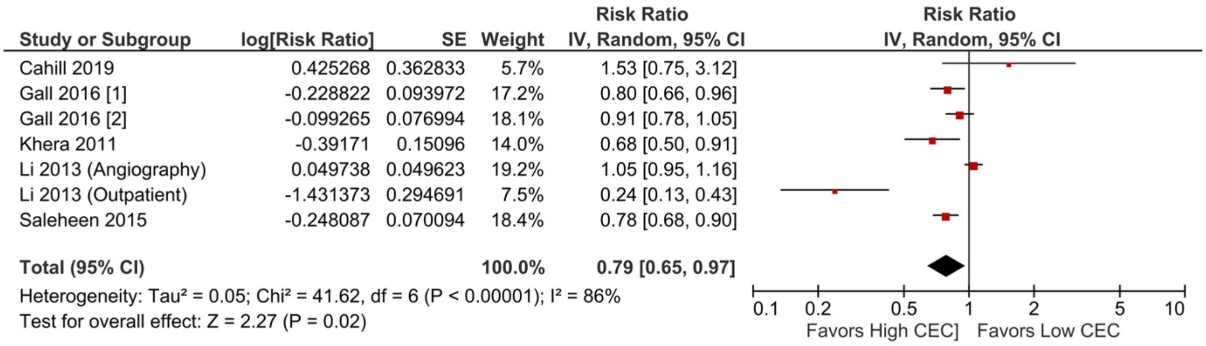

Figure S4. Atherosclerotic Cardiovascular Disease: Per SD Increment of CEC (HR) (2 studies)

Study or Subgroup	log[Hazard Ratio]	SE	Weight	Hazard Ratio IV, Random, 95% CI	Hazard Ratio IV, Random, 95% CI
Kopecky 2016 [1]	-0.040822	0.047475	56.7%	0.96 [0.87, 1.05]	
Rohatgi 2014 [1]	-0.287682	0.102037	43.3%	0.75 [0.61, 0.92]	
**Total (95% CI)**			**100.0%**	**0.86 [0.68, 1.10]**	

Heterogeneity: Tau² = 0.02; Chi² = 4.81, df = 1 (P = 0.03); I² = 79%

Test for overall effect: Z = 1.21 (P = 0.23)

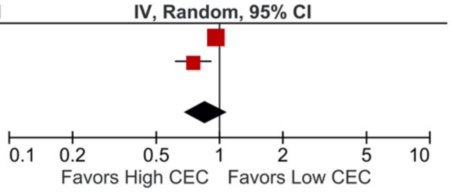

Figure S5. Atherosclerotic Cardiovascular Disease: Per SD Increment of CEC (Adjusted HR) (6 studies)

Study or Subgroup	log[Hazard Ratio]	SE	Weight	Hazard Ratio IV, Random, 95% CI
Chindhy 2018 (CKD) [1]	0.262364	0.124239	16.2%	1.30 [1.02, 1.66]
Chindhy 2018 (Non-CKD) [1]	-0.223144	0.10474	17.1%	0.80 [0.65, 0.98]
Kopecky 2016 [1]	-0.020203	0.036505	19.6%	0.98 [0.91, 1.05]
Mody 2016 (FHx of MI)	-0.579818	0.173614	13.7%	0.56 [0.40, 0.79]
Mody 2016 (Overall)	-0.510826	0.117228	16.5%	0.60 [0.48, 0.75]
Rohatgi 2014 [1]	-0.385662	0.108032	17.0%	0.68 [0.55, 0.84]
**Total (95% CI)**			**100.0%**	**0.80 [0.64, 1.00]**

Heterogeneity: Tau² = 0.07; Chi² = 41.58, df = 5 (P < 0.00001); I² = 88%
Test for overall effect: Z = 2.00 (P = 0.05)

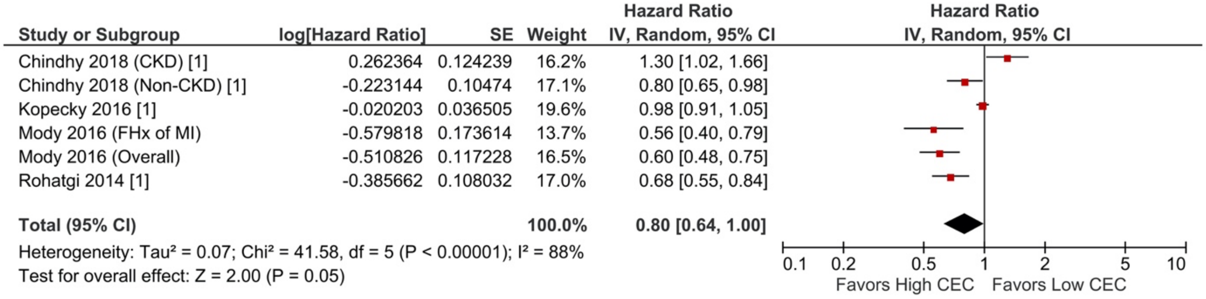

Figure S6. Atherosclerotic Cardiovascular Disease: Per SD Increment of CEC (OR) (5 studies)

Study or Subgroup	log[Odds Ratio]	SE	Weight	Odds Ratio IV, Random, 95% CI
Ebtehaj 2019	-0.301105	0.079391	23.1%	0.74 [0.63, 0.86]
Ishikawa 2015	-1.469676	0.628853	2.2%	0.23 [0.07, 0.79]
Khera 2017	-0.061875	0.091311	22.0%	0.94 [0.79, 1.12]
Saleheen 2015	-0.356675	0.050466	25.5%	0.70 [0.63, 0.77]
Tejera-Segura 2017	-0.051293	0.018707	27.1%	0.95 [0.92, 0.99]
**Total (95% CI)**			**100.0%**	**0.80 [0.66, 0.97]**

Heterogeneity: Tau² = 0.03; Chi² = 43.97, df = 4 (P < 0.00001); I² = 91%
Test for overall effect: Z = 2.27 (P = 0.02)

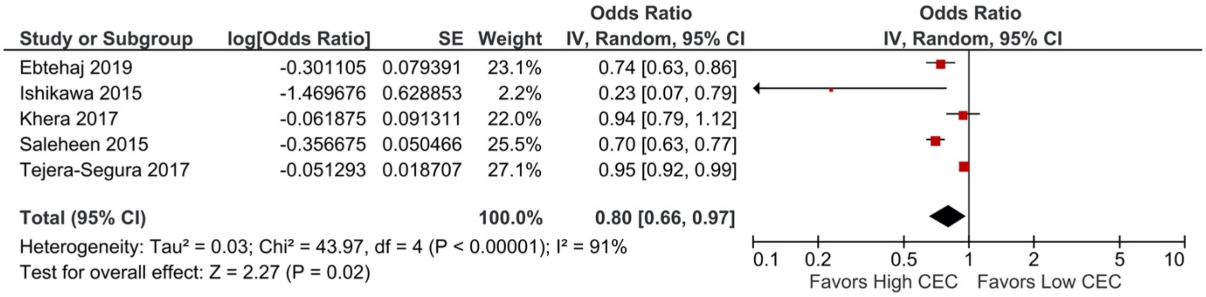

Figure S7. Atherosclerotic Cardiovascular Disease: Per SD Increment of CEC (Adjusted OR) (8 studies)

Study or Subgroup	log[Odds Ratio]	SE	Weight	Odds Ratio IV, Random, 95% CI
Ebtehaj 2019	-0.356675	0.094483	13.3%	0.70 [0.58, 0.84]
Ishikawa 2015	-1.469676	0.711248	0.6%	0.23 [0.06, 0.93]
Khera 2011	-0.287682	0.090989	13.6%	0.75 [0.63, 0.90]
Khera 2017	-0.116534	0.108116	11.9%	0.89 [0.72, 1.10]
Ogura 2016	-0.051293	0.024314	20.6%	0.95 [0.91, 1.00]
Saleheen 2015	-0.223144	0.064111	16.7%	0.80 [0.71, 0.91]
Tejera-Segura 2017	-0.061875	0.024574	20.5%	0.94 [0.90, 0.99]
Zhang 2016 [3]	-1.386294	0.303465	2.9%	0.25 [0.14, 0.45]
**Total (95% CI)**			**100.0%**	**0.81 [0.73, 0.90]**

Heterogeneity: $Tau^2 = 0.01$; $Chi^2 = 42.90$, $df = 7$ ($P < 0.00001$); $I^2 = 84\%$
Test for overall effect: $Z = 3.76$ ($P = 0.0002$)

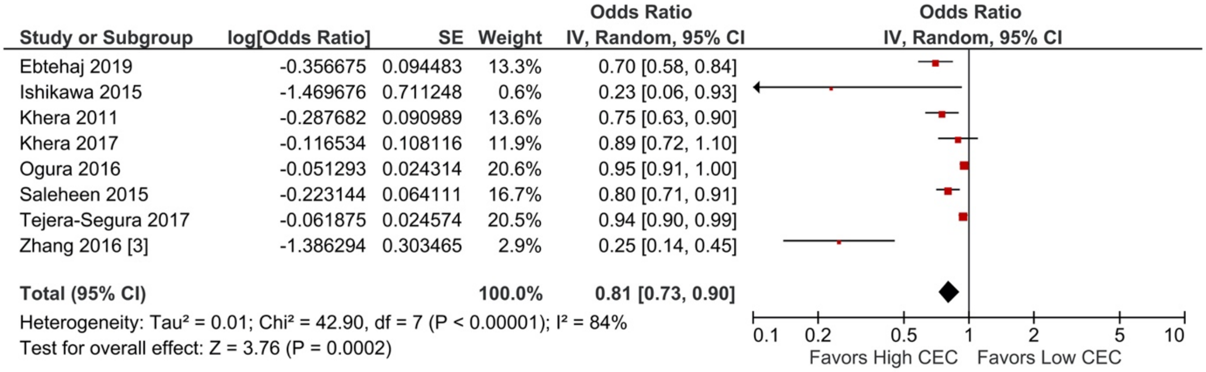

Figure S8. Death from All Causes: High CEC vs. Low CEC (RR) (3 studies)

Study or Subgroup	log[Risk Ratio]	SE	Weight	Risk Ratio IV, Random, 95% CI
Annema 2016 [1]	-0.006079	0.204685	33.4%	0.99 [0.67, 1.48]
Kopecky 2016 [2]	0.075563	0.072123	35.8%	1.08 [0.94, 1.24]
Liu 2016 [1]	-1.669157	0.294631	30.8%	0.19 [0.11, 0.34]
**Total (95% CI)**			**100.0%**	**0.61 [0.27, 1.41]**

Heterogeneity: Tau² = 0.50; Chi² = 33.09, df = 2 (P < 0.00001); I² = 94%
Test for overall effect: Z = 1.15 (P = 0.25)

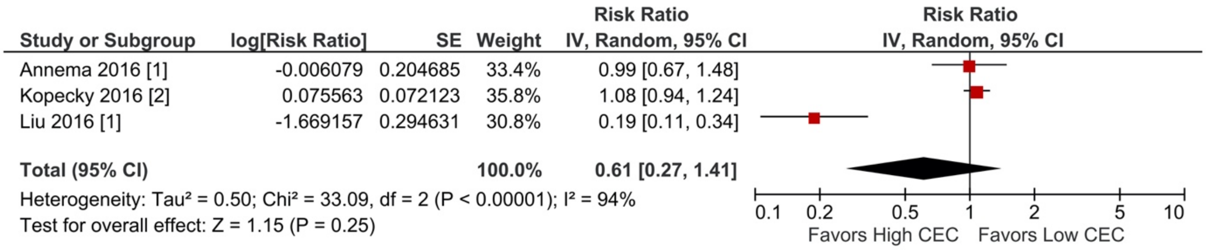

Figure S9. Death from All Causes: Per SD Increment of CEC (HR) (4 studies)

Study or Subgroup	log[Hazard Ratio]	SE	Weight	Hazard Ratio IV, Random, 95% CI
Annema 2016 [1]	-0.094311	0.103435	26.9%	0.91 [0.74, 1.11]
Guerin 2018	-0.415515	0.068952	30.3%	0.66 [0.58, 0.76]
Javaheri 2016	-0.446287	0.303465	10.7%	0.64 [0.35, 1.16]
Kopecky 2016 [2]	-0.040822	0.045057	32.1%	0.96 [0.88, 1.05]
**Total (95% CI)**			**100.0%**	**0.81 [0.64, 1.02]**

Heterogeneity: Tau² = 0.04; Chi² = 21.94, df = 3 (P < 0.0001); I² = 86%
Test for overall effect: Z = 1.76 (P = 0.08)

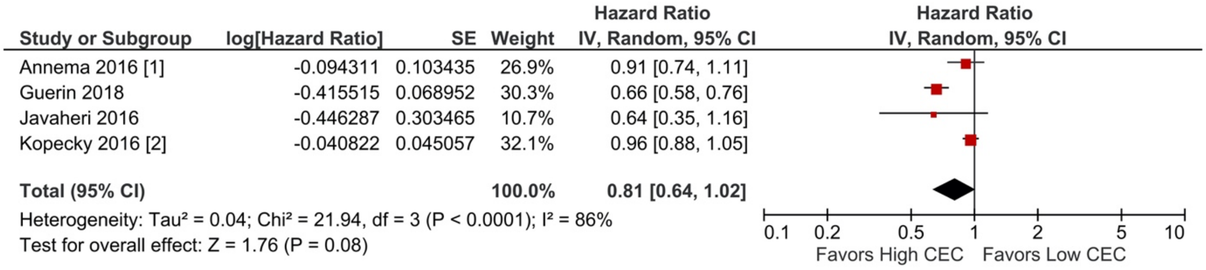

Figure S10. Death from All Causes: Per SD Increment of CEC (Adjusted HR) (5 studies)

Study or Subgroup	log[Hazard Ratio]	SE	Weight	Hazard Ratio IV, Random, 95% CI
Annema 2016 [1]	-0.083382	0.168973	23.8%	0.92 [0.66, 1.28]
Guerin 2018	-0.235722	0.092914	30.8%	0.79 [0.66, 0.95]
Javaheri 2016	-1.347074	0.445214	8.1%	0.26 [0.11, 0.62]
Kopecky 2016 [2]	-0.01005	0.028231	34.8%	0.99 [0.94, 1.05]
Liu 2016 [2]	-2.302585	0.871869	2.5%	0.10 [0.02, 0.55]
**Total (95% CI)**			**100.0%**	**0.77 [0.58, 1.02]**

Heterogeneity: Tau² = 0.06; Chi² = 20.97, df = 4 (P = 0.0003); I² = 81%

Test for overall effect: Z = 1.83 (P = 0.07)

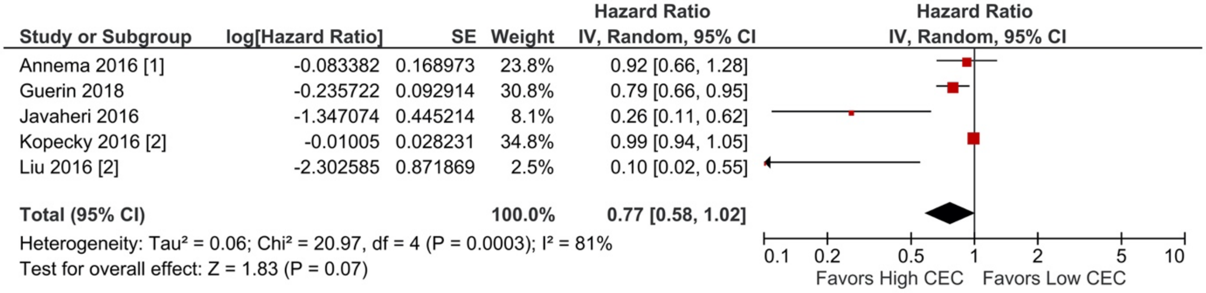

Figure S11. Death from Cardiovascular Causes: High CEC vs. Low CEC (RR) (4 studies)

Study or Subgroup	log[Risk Ratio]	SE	Weight	Risk Ratio IV, Random, 95% CI
Annema 2016 [2]	0.356826	0.306301	26.6%	1.43 [0.78, 2.60]
Liu 2016 [2]	-1.985915	0.397312	25.4%	0.14 [0.06, 0.30]
Rohatgi 2014 [2]	0.105361	0.455839	24.6%	1.11 [0.45, 2.71]
Zhang 2016 [2]	-1.484907	0.541087	23.3%	0.23 [0.08, 0.65]
**Total (95% CI)**			**100.0%**	**0.48 [0.14, 1.62]**

Heterogeneity: Tau² = 1.35; Chi² = 26.87, df = 3 (P < 0.00001); I² = 89%
Test for overall effect: Z = 1.18 (P = 0.24)

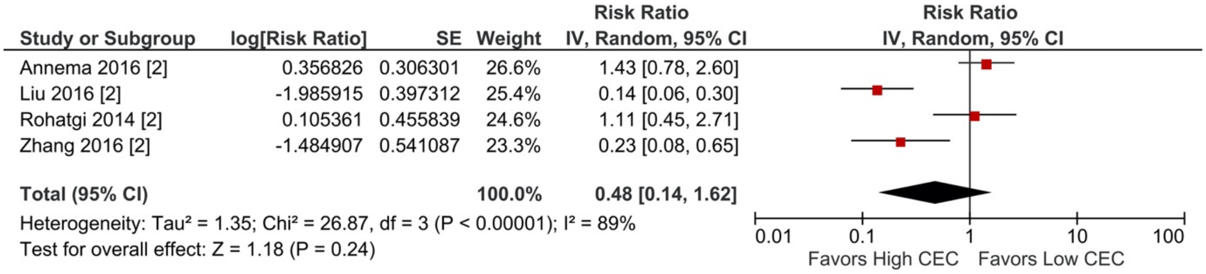

Figure S12. Death from Cardiovascular Causes: Per SD Increment of CEC (Adjusted HR) (4 studies)

Study or Subgroup	log[Hazard Ratio]	SE	Weight	Hazard Ratio IV, Random, 95% CI
Annema 2016 [2]	0.231112	0.209925	28.9%	1.26 [0.83, 1.90]
Chindhy 2018 (CKD) [2]	0.350657	0.138677	34.8%	1.42 [1.08, 1.86]
Chindhy 2018 (Non-CKD) [2]	-0.083382	0.162703	32.9%	0.92 [0.67, 1.27]
Liu 2016 [2]	-2.525729	1.076405	3.4%	0.08 [0.01, 0.66]
**Total (95% CI)**			**100.0%**	**1.08 [0.72, 1.62]**

Heterogeneity: Tau² = 0.10; Chi² = 10.47, df = 3 (P = 0.01); I² = 71%
Test for overall effect: Z = 0.37 (P = 0.71)

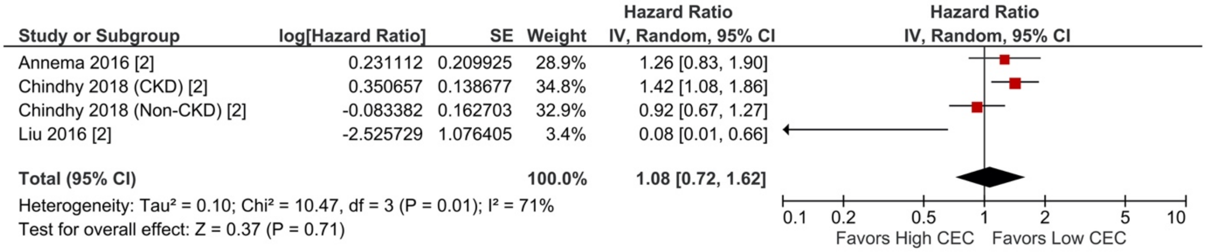

Figure S13. Adverse Cardiovascular Event: Mean Difference in CEC (6 studies)

	Case			Control				Mean Difference
**Study or Subgroup**	**Mean**	**SD**	**Total**	**Mean**	**SD**	**Total**	**Weight**	**IV, Random, 95% CI**
Cahill 2019	0.97	0.14	701	0.99	0.14	696	19.9%	-0.02 [-0.03, -0.01]
Ebtehaj 2019	0.93	0.29	351	1.01	0.24	354	16.4%	-0.08 [-0.12, -0.04]
Guerin 2018	0.762	0.14	239	0.818	0.138	1370	19.4%	-0.06 [-0.08, -0.04]
Ishikawa 2015	0.86	0.26	182	1.02	0.38	72	8.3%	-0.16 [-0.26, -0.06]
Javaheri 2016	0.89	0.03	15	0.98	0.03	20	19.3%	-0.09 [-0.11, -0.07]
Ogura 2016	0.79	0.15	76	0.92	0.11	151	16.7%	-0.13 [-0.17, -0.09]
**Total (95% CI)**			**1564**			**2663**	**100.0%**	**-0.08 [-0.12, -0.04]**

Heterogeneity: Tau² = 0.00; Chi² = 54.63, df = 5 (P < 0.00001); I² = 91%
Test for overall effect: Z = 4.43 (P < 0.00001)

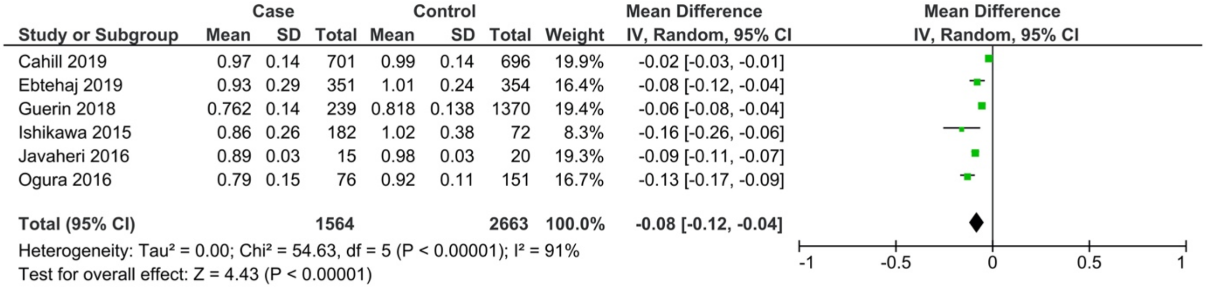

Figure S14. Atherosclerotic Cardiovascular Disease: Mean Difference in CEC (4 studies)

	Case			Control				Mean Difference	Mean Difference
**Study or Subgroup**	**Mean**	**SD**	**Total**	**Mean**	**SD**	**Total**	**Weight**	**IV, Random, 95% CI**	**IV, Random, 95% CI**
Cahill 2019	0.97	0.14	701	0.99	0.14	696	28.8%	-0.02 [-0.03, -0.01]	
Ebtehaj 2019	0.93	0.29	351	1.01	0.24	354	26.5%	-0.08 [-0.12, -0.04]	
Ishikawa 2015	0.86	0.26	182	1.02	0.38	72	18.1%	-0.16 [-0.26, -0.06]	
Ogura 2016	0.79	0.15	76	0.92	0.11	151	26.6%	-0.13 [-0.17, -0.09]	
**Total (95% CI)**			**1310**			**1273**	**100.0%**	**-0.09 [-0.16, -0.02]**	

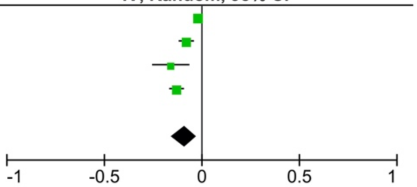

Heterogeneity: Tau² = 0.00; Chi² = 38.65, df = 3 (P < 0.00001); I² = 92%
Test for overall effect: Z = 2.69 (P = 0.007)

Figure S15. Death from All Causes: Mean Difference in CEC (2 studies)

	Case			Control				Mean Difference
**Study or Subgroup**	**Mean**	**SD**	**Total**	**Mean**	**SD**	**Total**	**Weight**	**IV, Random, 95% CI**
Guerin 2018	0.762	0.14	239	0.818	0.138	1370	50.4%	-0.06 [-0.08, -0.04]
Javaheri 2016	0.89	0.03	15	0.98	0.03	20	49.6%	-0.09 [-0.11, -0.07]
**Total (95% CI)**			**254**			**1390**	**100.0%**	**-0.07 [-0.11, -0.04]**

Heterogeneity: Tau² = 0.00; Chi² = 5.75, df = 1 (P = 0.02); I² = 83%
Test for overall effect: Z = 4.29 (P < 0.0001)

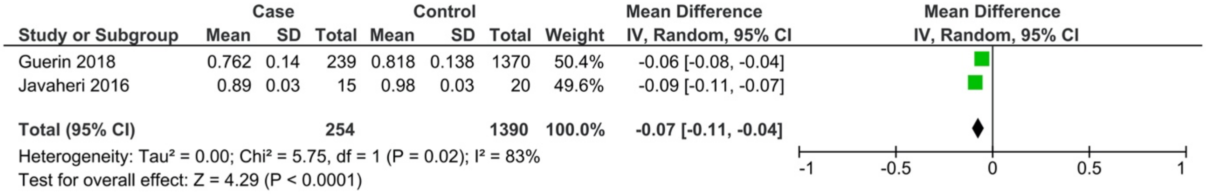

Figure S16. Funnel Plot of Adverse Cardiovascular Event: High CEC vs. Low CEC (RR) (14 studies)

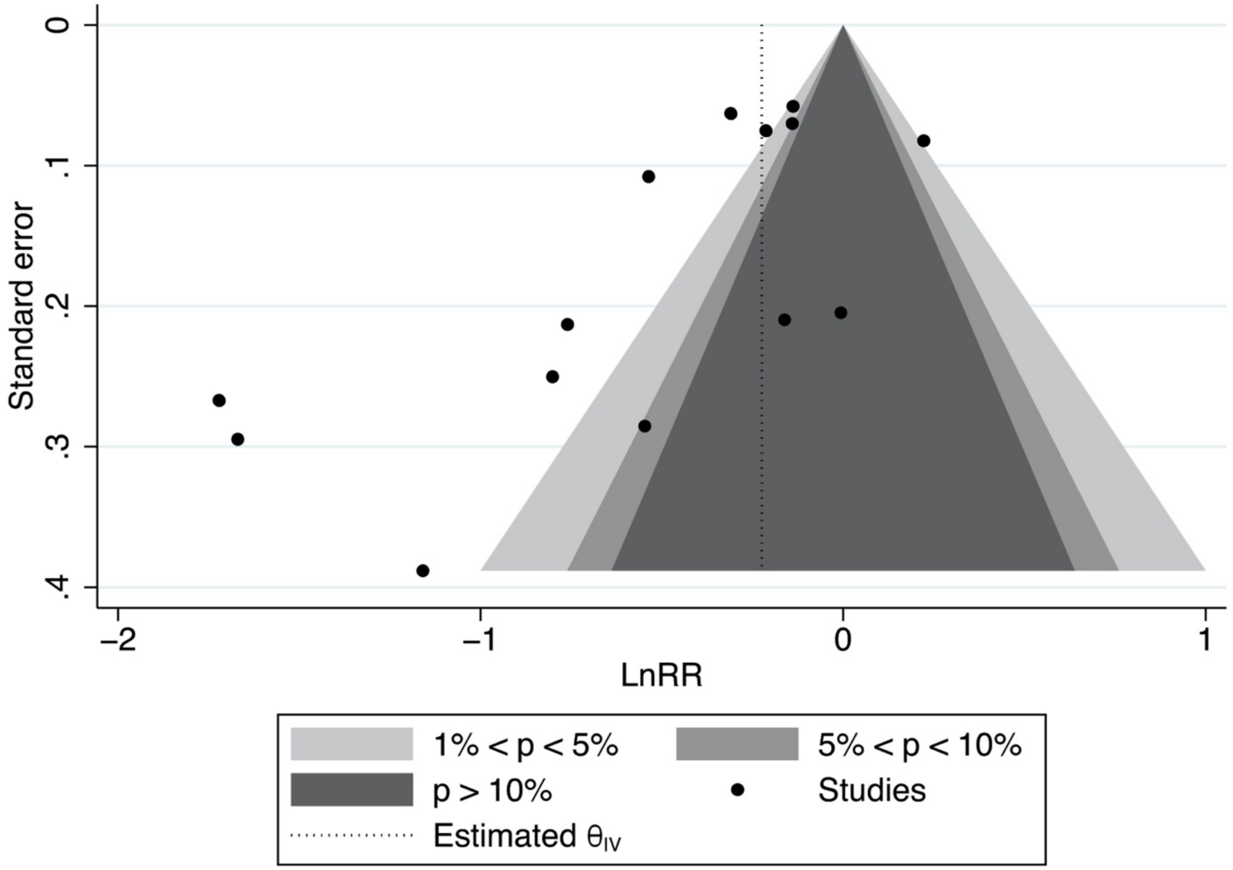

Figure S17. Funnel Plot of Adverse Cardiovascular Event: Per SD Increment of CEC (Adjusted HR) (10 studies)

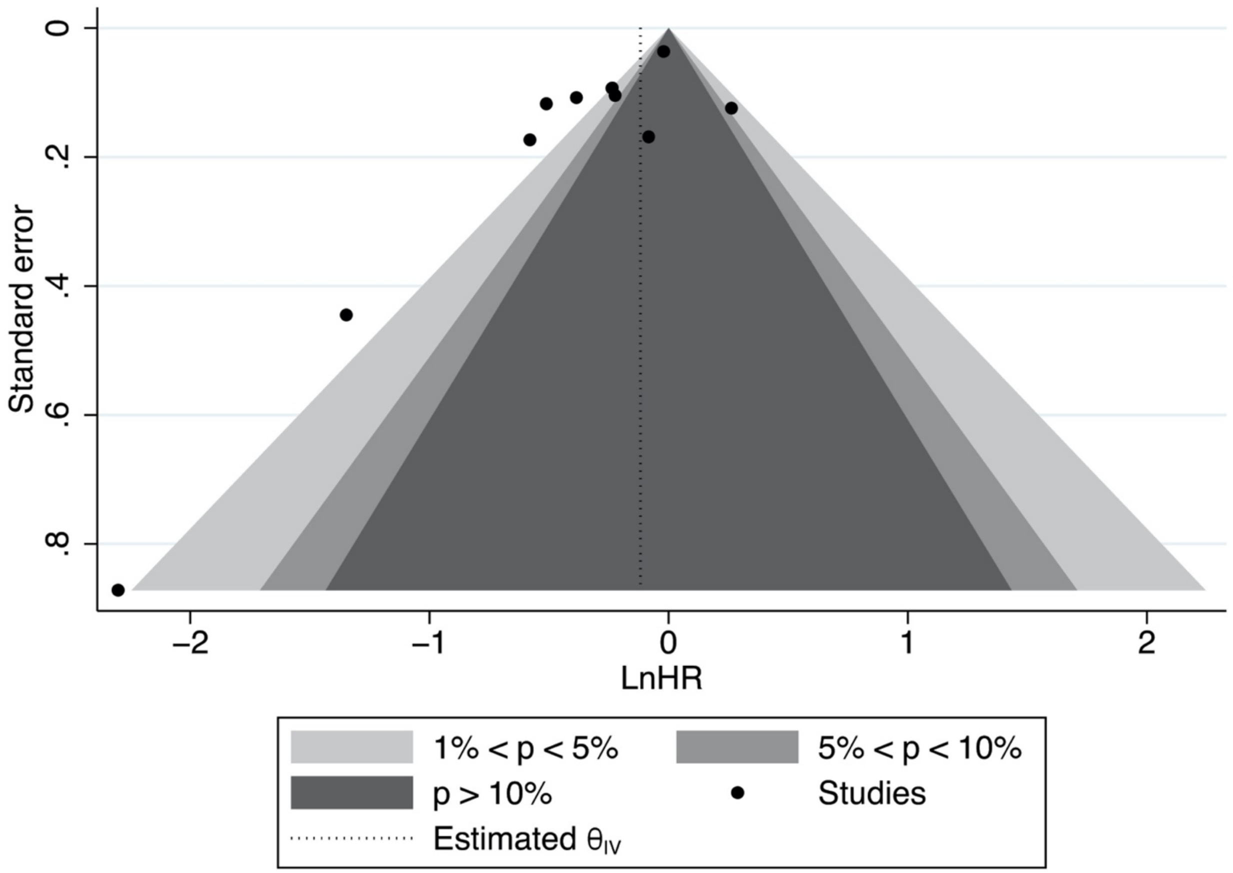

Figure S18. Funnel Plot of Atherosclerotic Cardiovascular Disease: High CEC vs. Low CEC (RR) (12 studies)

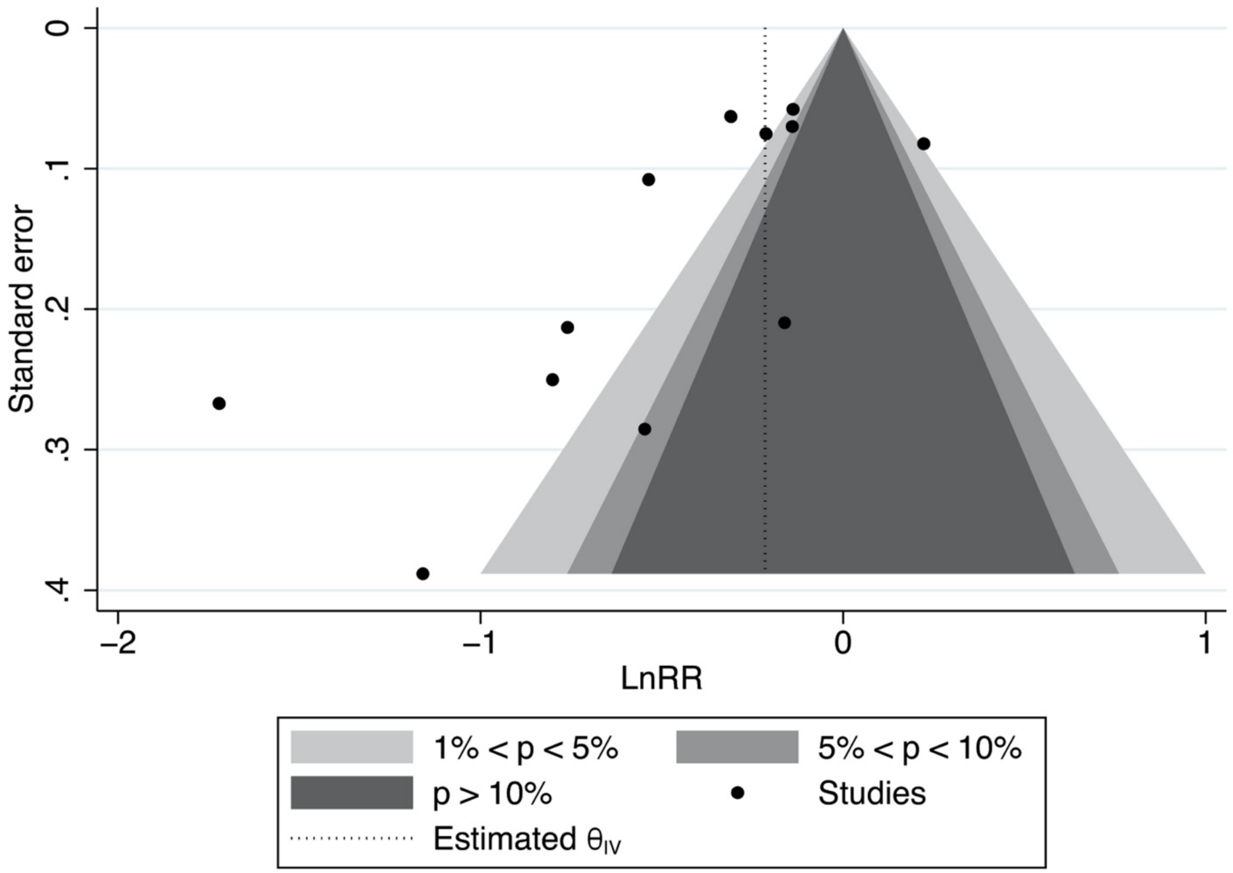

Figure S19. Subgroup Analysis of Adverse Cardiovascular Event: High CEC vs. Low CEC (RR) (14 studies)

Study or Subgroup	log[Risk Ratio]	SE	Weight	Risk Ratio IV, Random, 95% CI
**6.1.1 Without Cardiovascular Risk Factors or Chronic Kidney Disease**				
Ebtehaj 2019	−0.536578	0.10772	8.6%	0.58 [0.47, 0.72]
Li 2013 (Angiography)	−0.138627	0.057943	9.3%	0.87 [0.78, 0.98]
Li 2013 (Outpatient)	−1.720713	0.26714	5.6%	0.18 [0.11, 0.30]
Mody 2016 (Overall)	−0.759724	0.213029	6.6%	0.47 [0.31, 0.71]
Rohatgi 2014 [1]	−0.800778	0.250081	5.9%	0.45 [0.28, 0.73]
Saleheen 2015	−0.309309	0.06307	9.3%	0.73 [0.65, 0.83]
**Subtotal (95% CI)**			**45.2%**	**0.54 [0.41, 0.71]**
Heterogeneity: $Tau^2 = 0.10$; $Chi^2 = 49.32$, df = 5 (P < 0.00001); $I^2 = 90\%$				
Test for overall effect: Z = 4.30 (P < 0.0001)				
**6.1.2 With Cardiovascular Risk Factors**				
Gall 2016 [1]	−0.212331	0.075257	9.1%	0.81 [0.70, 0.94]
Gall 2016 [2]	−0.140741	0.070033	9.2%	0.87 [0.76, 1.00]
Liu 2016 [1]	−1.669157	0.294631	5.1%	0.19 [0.11, 0.34]
Mody 2016 (FHx of MI)	−0.546836	0.285258	5.2%	0.58 [0.33, 1.01]
Zhang 2016 [1]	−1.159485	0.388176	3.8%	0.31 [0.15, 0.67]
**Subtotal (95% CI)**			**32.4%**	**0.54 [0.38, 0.78]**
Heterogeneity: $Tau^2 = 0.12$; $Chi^2 = 32.44$, df = 4 (P < 0.00001); $I^2 = 88\%$				
Test for overall effect: Z = 3.28 (P = 0.001)				
**6.1.3 With Chronic Kidney Disease**				
Annema 2016 [1]	−0.006079	0.204685	6.7%	0.99 [0.67, 1.48]
Bauer 2017	−0.161663	0.209802	6.6%	0.85 [0.56, 1.28]
Kopecky 2016 [1]	0.223088	0.082374	9.0%	1.25 [1.06, 1.47]
**Subtotal (95% CI)**			**22.4%**	**1.08 [0.86, 1.38]**
Heterogeneity: $Tau^2 = 0.02$; $Chi^2 = 3.58$, df = 2 (P = 0.17); $I^2 = 44\%$				
Test for overall effect: Z = 0.67 (P = 0.50)				
**Total (95% CI)**			**100.0%**	**0.63 [0.52, 0.76]**
Heterogeneity: $Tau^2 = 0.10$; $Chi^2 = 118.72$, df = 13 (P < 0.00001); $I^2 = 89\%$				
Test for overall effect: Z = 4.77 (P < 0.00001)				
Test for subgroup differences: $Chi^2 = 17.59$, df = 2 (P = 0.0002), $I^2 = 88.6\%$				

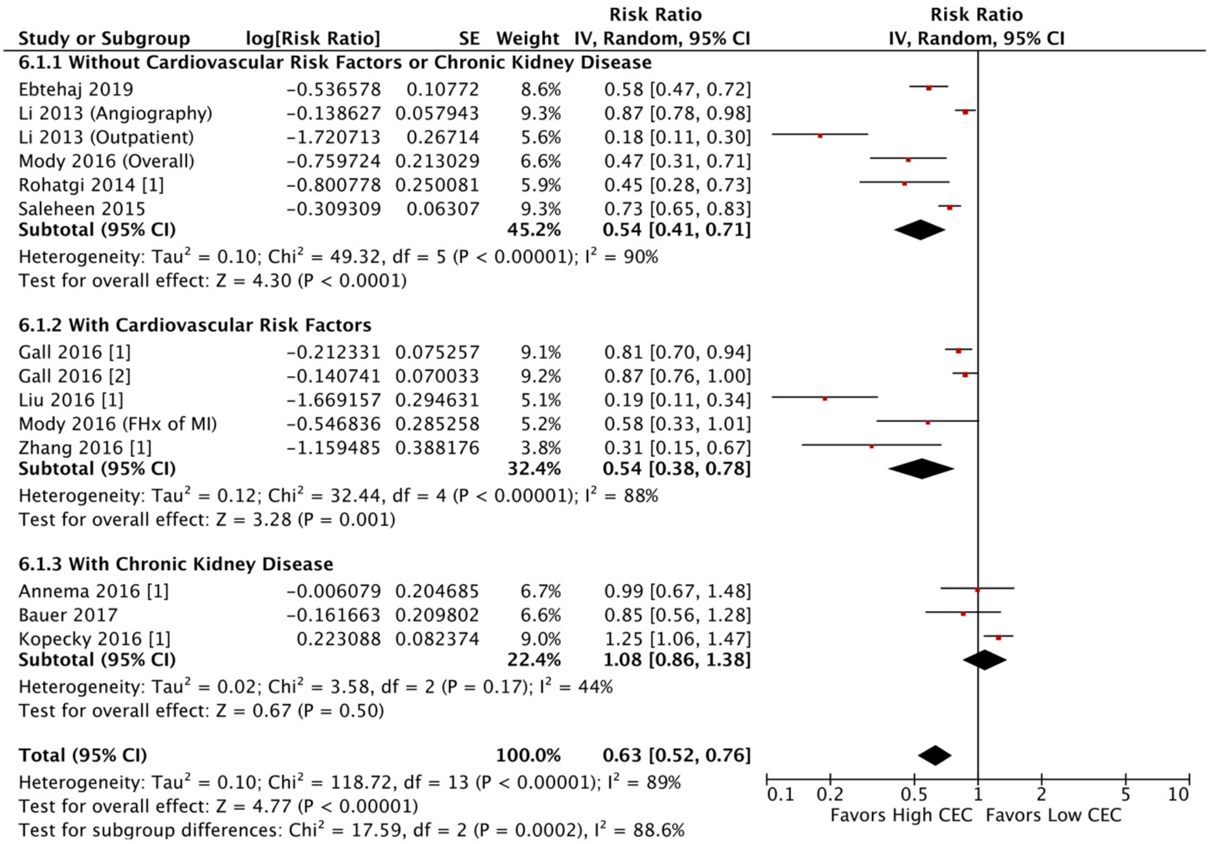

Figure S20. Subgroup Analysis of Adverse Cardiovascular Event: Per SD Increment of CEC (Adjusted HR) (10 studies)

Study or Subgroup	log[Hazard Ratio]	SE	Weight	Hazard Ratio IV, Random, 95% CI
**6.2.1 Without Cardiovascular Risk Factors or Chronic Kidney Disease**				
Chindhy 2018 (Non–CKD) [1]	−0.223144	0.10474	12.4%	0.80 [0.65, 0.98]
Mody 2016 (Overall)	−0.510826	0.117228	12.0%	0.60 [0.48, 0.75]
Rohatgi 2014 [1]	−0.385662	0.108032	12.3%	0.68 [0.55, 0.84]
**Subtotal (95% CI)**			**36.7%**	**0.69 [0.59, 0.82]**
Heterogeneity: $Tau^2 = 0.01$; $Chi^2 = 3.42$, df = 2 (P = 0.18); $I^2 = 41\%$				
Test for overall effect: Z = 4.43 (P < 0.00001)				
**6.2.2 With Cardiovascular Risk Factors**				
Guerin 2018	−0.235722	0.092914	12.8%	0.79 [0.66, 0.95]
Javaheri 2016	−1.347074	0.445214	3.5%	0.26 [0.11, 0.62]
Liu 2016 [1]	−2.302585	0.871869	1.1%	0.10 [0.02, 0.55]
Mody 2016 (FHx of MI)	−0.579818	0.173614	9.9%	0.56 [0.40, 0.79]
**Subtotal (95% CI)**			**27.3%**	**0.49 [0.29, 0.82]**
Heterogeneity: $Tau^2 = 0.17$; $Chi^2 = 13.27$, df = 3 (P = 0.004); $I^2 = 77\%$				
Test for overall effect: Z = 2.74 (P = 0.006)				
**6.2.3 With Chronic Kidney Disease**				
Annema 2016 [1]	−0.083382	0.168973	10.0%	0.92 [0.66, 1.28]
Chindhy 2018 (CKD) [1]	0.262364	0.124239	11.7%	1.30 [1.02, 1.66]
Kopecky 2016 [1]	−0.020203	0.036505	14.3%	0.98 [0.91, 1.05]
**Subtotal (95% CI)**			**36.0%**	**1.05 [0.87, 1.27]**
Heterogeneity: $Tau^2 = 0.02$; $Chi^2 = 5.01$, df = 2 (P = 0.08); $I^2 = 60\%$				
Test for overall effect: Z = 0.50 (P = 0.62)				
**Total (95% CI)**			**100.0%**	**0.76 [0.63, 0.91]**
Heterogeneity: $Tau^2 = 0.06$; $Chi^2 = 57.55$, df = 9 (P < 0.00001); $I^2 = 84\%$				
Test for overall effect: Z = 2.90 (P = 0.004)				
Test for subgroup differences: $Chi^2 = 14.58$, df = 2 (P = 0.0007), $I^2 = 86.3\%$				

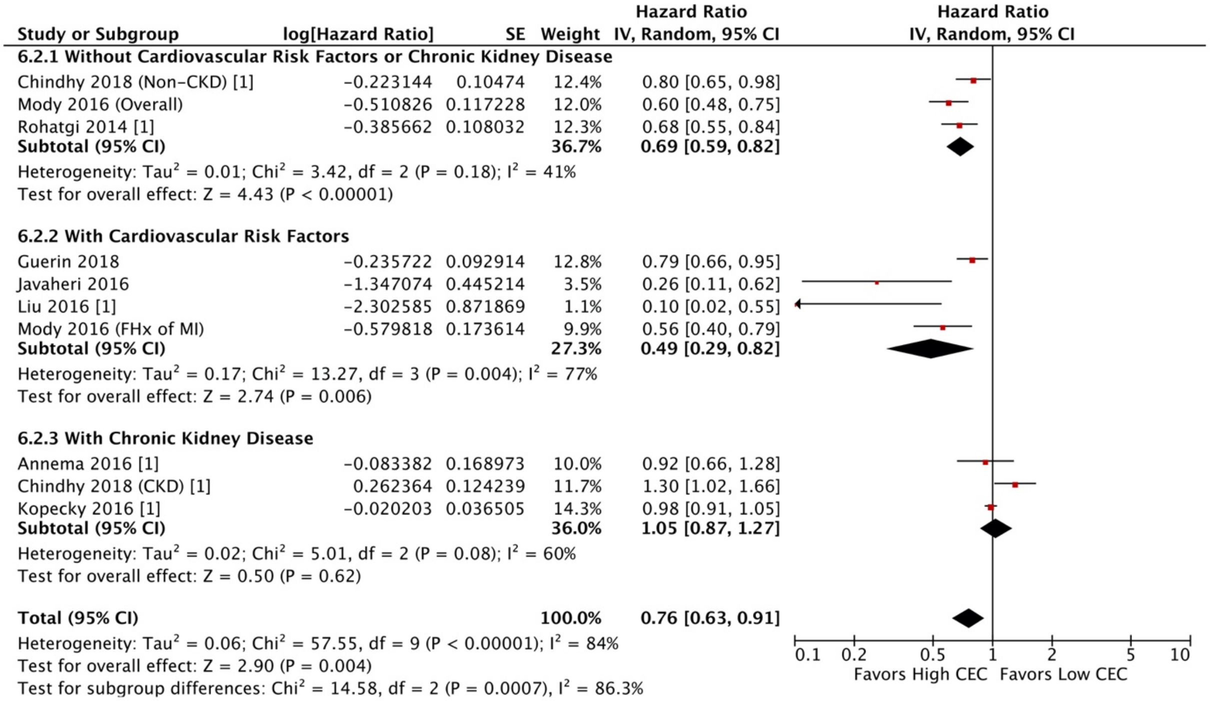

Figure S21. Subgroup Analysis of Atherosclerotic Cardiovascular Disease: High CEC vs. Low CEC (RR) (12 studies)

Study or Subgroup	log[Risk Ratio]	SE	Weight	Risk Ratio IV, Random, 95% CI
**6.3.1 Without Cardiovascular Risk Factors or Chronic Kidney Disease**				
Ebtehaj 2019	−0.536578	0.10772	9.9%	0.58 [0.47, 0.72]
Li 2013 (Angiography)	−0.138627	0.057943	10.9%	0.87 [0.78, 0.98]
Li 2013 (Outpatient)	−1.720713	0.26714	6.0%	0.18 [0.11, 0.30]
Mody 2016 (Overall)	−0.759724	0.213029	7.2%	0.47 [0.31, 0.71]
Rohatgi 2014 [1]	−0.800778	0.250081	6.3%	0.45 [0.28, 0.73]
Saleheen 2015	−0.309309	0.06307	10.9%	0.73 [0.65, 0.83]
**Subtotal (95% CI)**			**51.3%**	**0.54 [0.41, 0.71]**
Heterogeneity: Tau² = 0.10; Chi² = 49.32, df = 5 (P < 0.00001); I² = 90%				
Test for overall effect: Z = 4.30 (P < 0.0001)				
**6.3.2 With Cardiovascular Risk Factors**				
Gall 2016 [1]	−0.212331	0.075257	10.6%	0.81 [0.70, 0.94]
Gall 2016 [2]	−0.140741	0.070033	10.7%	0.87 [0.76, 1.00]
Mody 2016 (FHx of MI)	−0.546836	0.285258	5.6%	0.58 [0.33, 1.01]
Zhang 2016 [1]	−1.159485	0.388176	3.9%	0.31 [0.15, 0.67]
**Subtotal (95% CI)**			**30.9%**	**0.75 [0.60, 0.93]**
Heterogeneity: Tau² = 0.02; Chi² = 8.33, df = 3 (P = 0.04); I² = 64%				
Test for overall effect: Z = 2.60 (P = 0.009)				
**6.3.3 With Chronic Kidney Disease**				
Bauer 2017	−0.161663	0.209802	7.3%	0.85 [0.56, 1.28]
Kopecky 2016 [1]	0.223088	0.082374	10.5%	1.25 [1.06, 1.47]
**Subtotal (95% CI)**			**17.8%**	**1.08 [0.75, 1.56]**
Heterogeneity: Tau² = 0.05; Chi² = 2.91, df = 1 (P = 0.09); I² = 66%				
Test for overall effect: Z = 0.42 (P = 0.67)				
**Total (95% CI)**			**100.0%**	**0.66 [0.55, 0.80]**
Heterogeneity: Tau² = 0.08; Chi² = 93.43, df = 11 (P < 0.00001); I² = 88%				
Test for overall effect: Z = 4.37 (P < 0.0001)				
Test for subgroup differences: Chi² = 9.03, df = 2 (P = 0.01), I² = 77.8%				

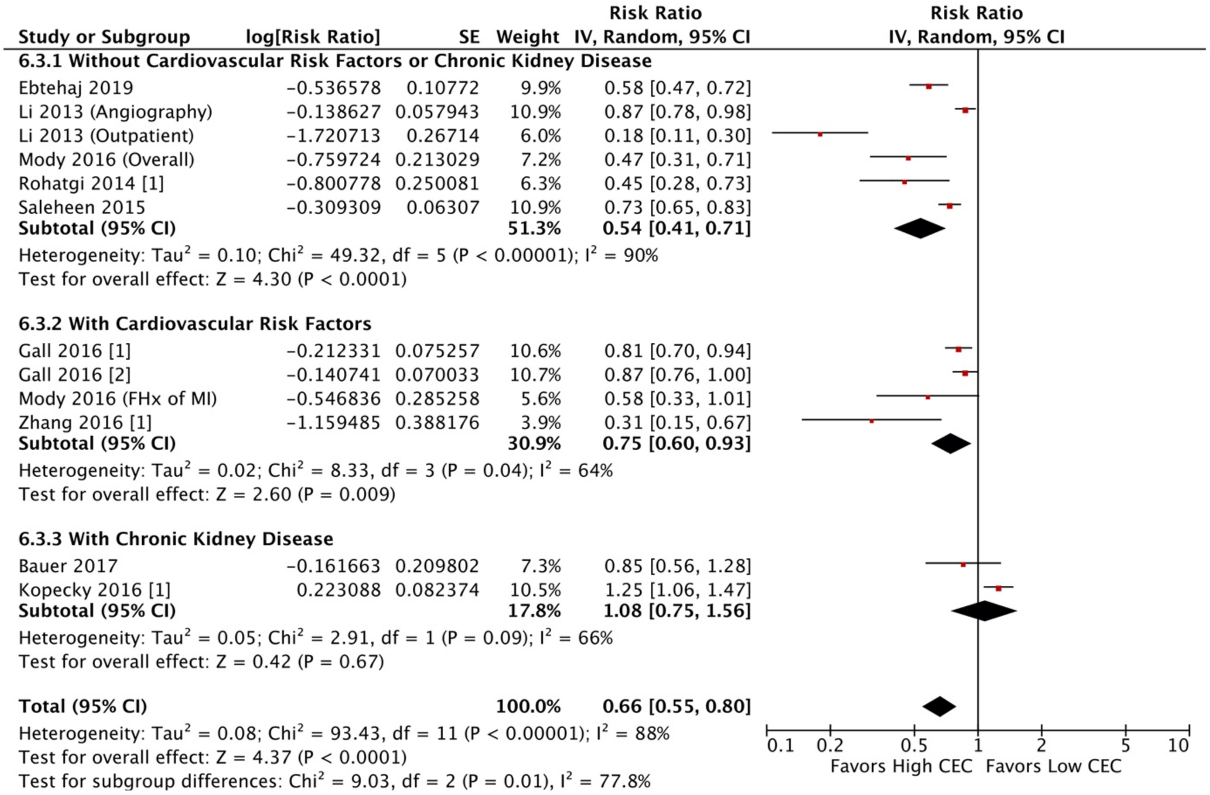

Figure S22. Adverse Cardiovascular Event Stratified by the Type of Cholesterol Donor: High CEC vs. Low CEC (RR) (14 studies)

Study or Subgroup	log[Risk Ratio]	SE	Weight	Risk Ratio IV, Random, 95% CI
**1.1.1 Donor: J774**				
Bauer 2017	−0.161663	0.209802	6.6%	0.85 [0.56, 1.28]
Ebtehaj 2019	−0.536578	0.10772	8.6%	0.58 [0.47, 0.72]
Gall 2016 [1]	−0.212331	0.075257	9.1%	0.81 [0.70, 0.94]
Gall 2016 [2]	−0.140741	0.070033	9.2%	0.87 [0.76, 1.00]
Li 2013 (Angiography)	−0.138627	0.057943	9.3%	0.87 [0.78, 0.98]
Li 2013 (Outpatient)	−1.720713	0.26714	5.6%	0.18 [0.11, 0.30]
Liu 2016 [1]	−1.669157	0.294631	5.1%	0.19 [0.11, 0.34]
Mody 2016 (FHx of MI)	−0.546836	0.285258	5.2%	0.58 [0.33, 1.01]
Mody 2016 (Overall)	−0.759724	0.213029	6.6%	0.47 [0.31, 0.71]
Rohatgi 2014 [1]	−0.800778	0.250081	5.9%	0.45 [0.28, 0.73]
Saleheen 2015	−0.309309	0.06307	9.3%	0.73 [0.65, 0.83]
Zhang 2016 [1]	−1.159485	0.388176	3.8%	0.31 [0.15, 0.67]
**Subtotal (95% CI)**			**84.2%**	**0.57 [0.47, 0.69]**
Heterogeneity: $Tau^2 = 0.08$; $Chi^2 = 83.59$, df = 11 ($P < 0.00001$); $I^2 = 87\%$				
Test for overall effect: Z = 5.69 ($P < 0.00001$)				
**1.1.2 Donor: THP-1**				
Annema 2016 [1]	−0.006079	0.204685	6.7%	0.99 [0.67, 1.48]
Kopecky 2016 [1]	0.223088	0.082374	9.0%	1.25 [1.06, 1.47]
**Subtotal (95% CI)**			**15.8%**	**1.20 [1.02, 1.42]**
Heterogeneity: $Tau^2 = 0.00$; $Chi^2 = 1.08$, df = 1 ($P = 0.30$); $I^2 = 7\%$				
Test for overall effect: Z = 2.17 ($P = 0.03$)				
**Total (95% CI)**			**100.0%**	**0.63 [0.52, 0.76]**
Heterogeneity: $Tau^2 = 0.10$; $Chi^2 = 118.72$, df = 13 ($P < 0.00001$); $I^2 = 89\%$				
Test for overall effect: Z = 4.77 ($P < 0.00001$)				
Test for subgroup differences: $Chi^2 = 32.69$, df = 1 ($P < 0.00001$), $I^2 = 96.9\%$				

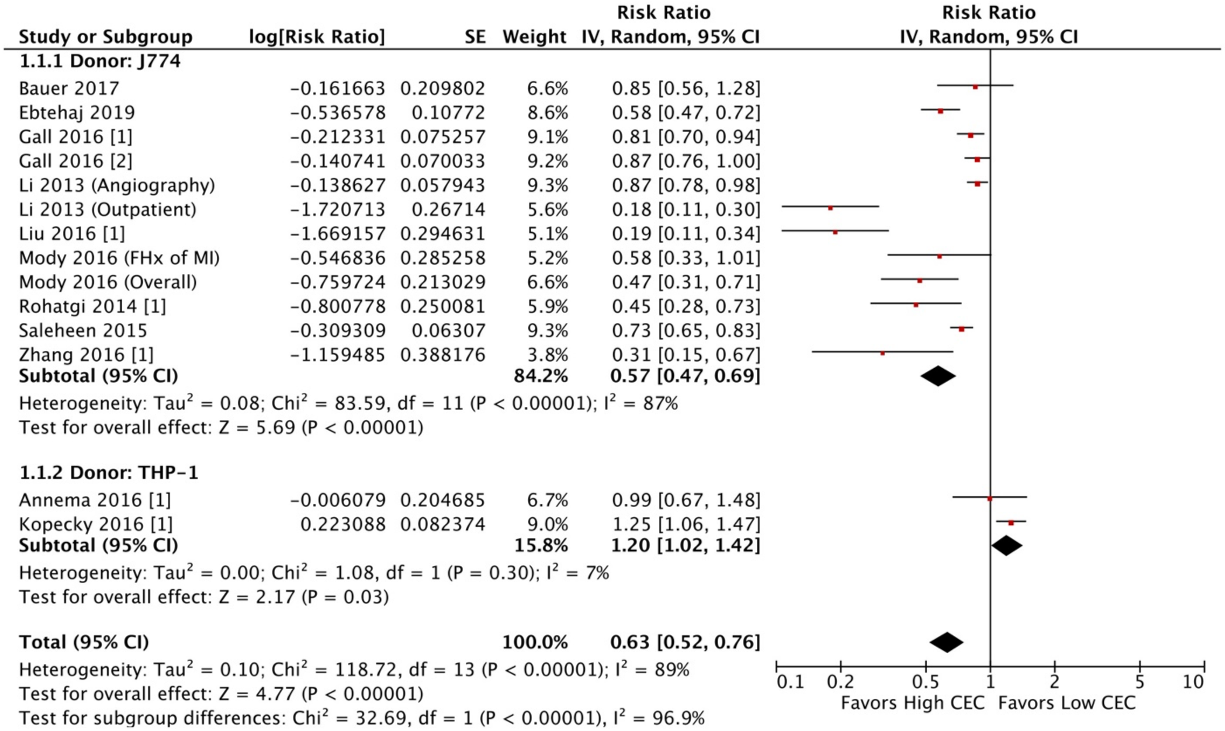

Figure S23. Adverse Cardiovascular Event Stratified by the Type of Cholesterol Donor: Per SD Increment of CEC (HR) (5 studies)

Study or Subgroup	log[Hazard Ratio]	SE	Weight	Hazard Ratio IV, Random, 95% CI
**1.2.1 Donor: J774**				
Javaheri 2016	−0.446287	0.303465	7.5%	0.64 [0.35, 1.16]
Rohatgi 2014 [1]	−0.287682	0.102037	21.2%	0.75 [0.61, 0.92]
**Subtotal (95% CI)**			**28.7%**	**0.74 [0.61, 0.89]**
Heterogeneity: $Tau^2 = 0.00$; $Chi^2 = 0.25$, df = 1 (P = 0.62); $I^2 = 0\%$				
Test for overall effect: Z = 3.14 (P = 0.002)				
**1.2.2 Donor: THP-1**				
Annema 2016 [2]	−0.094311	0.103435	21.1%	0.91 [0.74, 1.11]
Guerin 2018	−0.415515	0.068952	24.3%	0.66 [0.58, 0.76]
Kopecky 2016 [1]	−0.040822	0.047475	26.0%	0.96 [0.87, 1.05]
**Subtotal (95% CI)**			**71.3%**	**0.83 [0.65, 1.07]**
Heterogeneity: $Tau^2 = 0.04$; $Chi^2 = 20.40$, df = 2 (P < 0.0001); $I^2 = 90\%$				
Test for overall effect: Z = 1.42 (P = 0.15)				
**Total (95% CI)**			**100.0%**	**0.80 [0.66, 0.97]**
Heterogeneity: $Tau^2 = 0.03$; $Chi^2 = 22.77$, df = 4 (P = 0.0001); $I^2 = 82\%$				
Test for overall effect: Z = 2.32 (P = 0.02)				
Test for subgroup differences: $Chi^2 = 0.55$, df = 1 (P = 0.46), $I^2 = 0\%$				

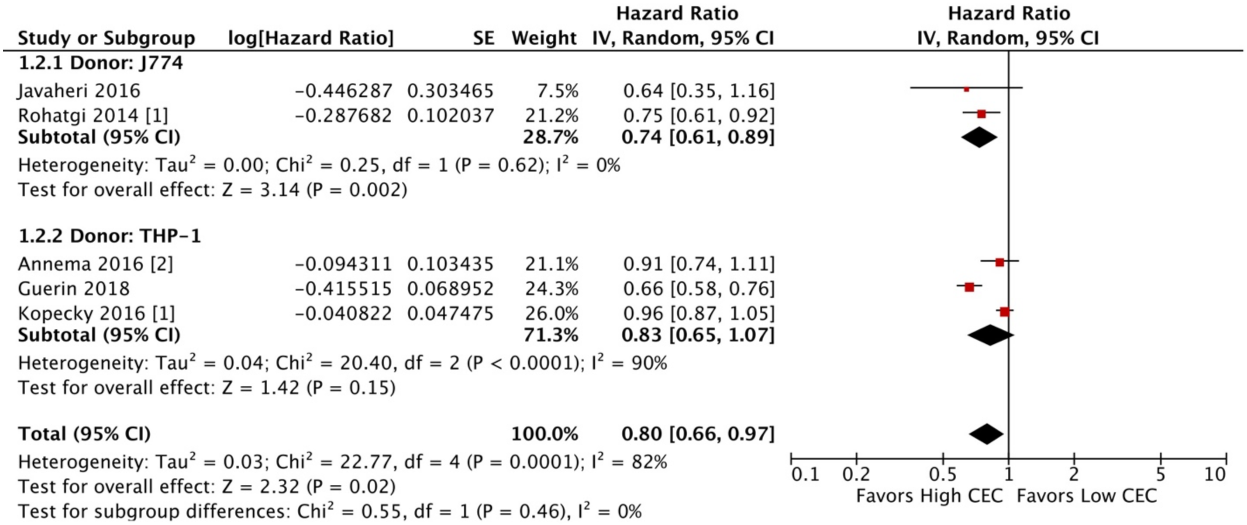

Figure S24. Adverse Cardiovascular Event Stratified by the Type of Cholesterol Donor: Per SD Increment of CEC (adjusted HR) (10 studies)

Study or Subgroup	log[Hazard Ratio]	SE	Weight	Hazard Ratio IV, Random, 95% CI
**1.3.1 Donor: J774**				
Chindhy 2018 (CKD) [1]	0.262364	0.124239	11.7%	1.30 [1.02, 1.66]
Chindhy 2018 (Non−CKD) [1]	−0.223144	0.10474	12.4%	0.80 [0.65, 0.98]
Javaheri 2016	−1.347074	0.445214	3.5%	0.26 [0.11, 0.62]
Liu 2016 [1]	−2.302585	0.871869	1.1%	0.10 [0.02, 0.55]
Mody 2016 (FHx of MI)	−0.579818	0.173614	9.9%	0.56 [0.40, 0.79]
Mody 2016 (Overall)	−0.510826	0.117228	12.0%	0.60 [0.48, 0.75]
Rohatgi 2014 [1]	−0.385662	0.108032	12.3%	0.68 [0.55, 0.84]
**Subtotal (95% CI)**			**62.9%**	**0.66 [0.49, 0.89]**
Heterogeneity: $Tau^2 = 0.12$; $Chi^2 = 38.26$, df = 6 (P < 0.00001); $I^2 = 84\%$				
Test for overall effect: Z = 2.71 (P = 0.007)				
**1.3.2 Donor: THP−1**				
Annema 2016 [1]	−0.083382	0.168973	10.0%	0.92 [0.66, 1.28]
Guerin 2018	−0.235722	0.092914	12.8%	0.79 [0.66, 0.95]
Kopecky 2016 [1]	−0.020203	0.036505	14.3%	0.98 [0.91, 1.05]
**Subtotal (95% CI)**			**37.1%**	**0.91 [0.78, 1.06]**
Heterogeneity: $Tau^2 = 0.01$; $Chi^2 = 4.70$, df = 2 (P = 0.10); $I^2 = 57\%$				
Test for overall effect: Z = 1.27 (P = 0.20)				
**Total (95% CI)**			**100.0%**	**0.76 [0.63, 0.91]**
Heterogeneity: $Tau^2 = 0.06$; $Chi^2 = 57.55$, df = 9 (P < 0.00001); $I^2 = 84\%$				
Test for overall effect: Z = 2.90 (P = 0.004)				
Test for subgroup differences: $Chi^2 = 3.39$, df = 1 (P = 0.07), $I^2 = 70.5\%$				

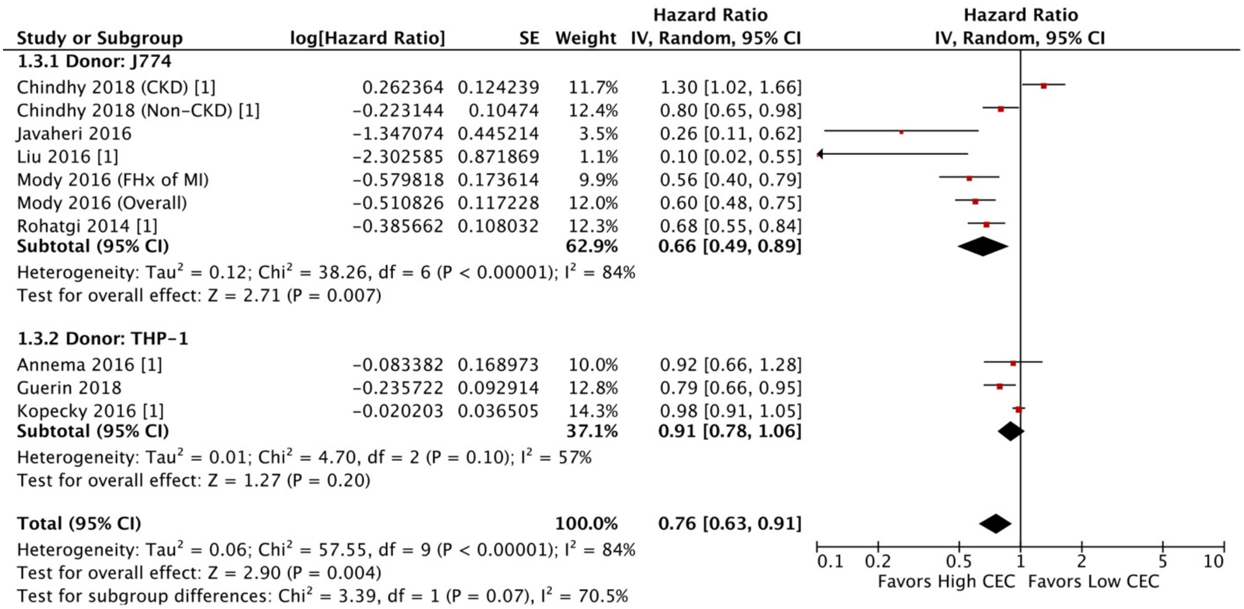

Figure S25. Adverse Cardiovascular Event Stratified by the Method of Labeling: High CEC vs. Low CEC (RR) (14 studies)

Study or Subgroup	log[Risk Ratio]	SE	Weight	Risk Ratio IV, Random, 95% CI	Risk Ratio IV, Random, 95% CI
**1.1.1 Label: [^{3}H]-Cholesterol**					
Annema 2016 [1]	−0.006079	0.204685	6.7%	0.99 [0.67, 1.48]	
Bauer 2017	−0.161663	0.209802	6.6%	0.85 [0.56, 1.28]	
Ebtehaj 2019	−0.536578	0.10772	8.6%	0.58 [0.47, 0.72]	
Gall 2016 [1]	−0.212331	0.075257	9.1%	0.81 [0.70, 0.94]	
Gall 2016 [2]	−0.140741	0.070033	9.2%	0.87 [0.76, 1.00]	
Kopecky 2016 [1]	0.223088	0.082374	9.0%	1.25 [1.06, 1.47]	
Li 2013 (Angiography)	−0.138627	0.057943	9.3%	0.87 [0.78, 0.98]	
Li 2013 (Outpatient)	−1.720713	0.26714	5.6%	0.18 [0.11, 0.30]	
Saleheen 2015	−0.309309	0.06307	9.3%	0.73 [0.65, 0.83]	
Zhang 2016 [1]	−1.159485	0.388176	3.8%	0.31 [0.15, 0.67]	
**Subtotal (95% CI)**			**77.2%**	**0.74 [0.61, 0.89]**	
Heterogeneity: $Tau^2 = 0.07$; $Chi^2 = 80.34$, df = 9 (P < 0.00001); $I^2 = 89\%$					
Test for overall effect: Z = 3.21 (P = 0.001)					
**1.1.2 Label: BODIPY-Cholesterol**					
Liu 2016 [1]	−1.669157	0.294631	5.1%	0.19 [0.11, 0.34]	
Mody 2016 (FHx of MI)	−0.546836	0.285258	5.2%	0.58 [0.33, 1.01]	
Mody 2016 (Overall)	−0.759724	0.213029	6.6%	0.47 [0.31, 0.71]	
Rohatgi 2014 [1]	−0.800778	0.250081	5.9%	0.45 [0.28, 0.73]	
**Subtotal (95% CI)**			**22.8%**	**0.40 [0.26, 0.61]**	
Heterogeneity: $Tau^2 = 0.13$; $Chi^2 = 8.94$, df = 3 (P = 0.03); $I^2 = 66\%$					
Test for overall effect: Z = 4.16 (P < 0.0001)					
**Total (95% CI)**			**100.0%**	**0.63 [0.52, 0.76]**	
Heterogeneity: $Tau^2 = 0.10$; $Chi^2 = 118.72$, df = 13 (P < 0.00001); $I^2 = 89\%$					
Test for overall effect: Z = 4.77 (P < 0.00001)					
Test for subgroup differences: $Chi^2 = 6.53$, df = 1 (P = 0.01), $I^2 = 84.7\%$					

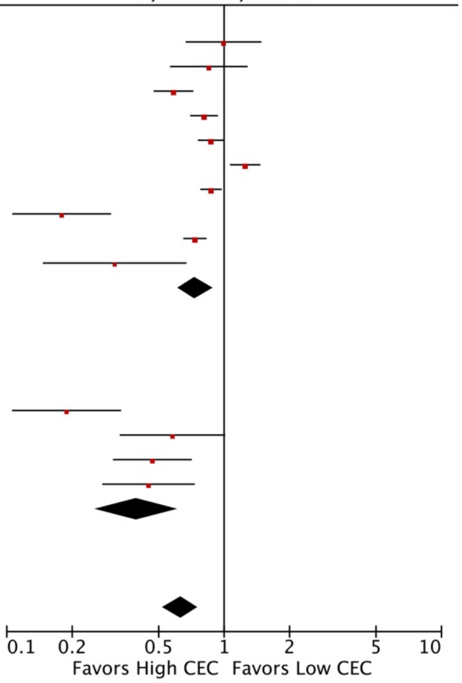

Figure S26. Adverse Cardiovascular Event Stratified by the Method of Labeling: Per SD Increment of CEC (HR) (5 studies)

Study or Subgroup	log[Hazard Ratio]	SE	Weight	Hazard Ratio IV, Random, 95% CI
**1.2.1 Label: [^{3}H]-Cholesterol**				
Annema 2016 [1]	−0.094311	0.103435	21.1%	0.91 [0.74, 1.11]
Guerin 2018	−0.415515	0.068952	24.3%	0.66 [0.58, 0.76]
Javaheri 2016	−0.446287	0.303465	7.5%	0.64 [0.35, 1.16]
Kopecky 2016 [1]	−0.040822	0.047475	26.0%	0.96 [0.87, 1.05]
**Subtotal (95% CI)**			**78.8%**	**0.81 [0.64, 1.02]**
Heterogeneity: $Tau^2 = 0.04$; $Chi^2 = 21.32$, df = 3 (P < 0.0001); $I^2 = 86\%$				
Test for overall effect: Z = 1.77 (P = 0.08)				
**1.2.2 Label: BODIPY-Cholesterol**				
Rohatgi 2014 [1]	−0.287682	0.102037	21.2%	0.75 [0.61, 0.92]
**Subtotal (95% CI)**			**21.2%**	**0.75 [0.61, 0.92]**
Heterogeneity: Not applicable				
Test for overall effect: Z = 2.82 (P = 0.005)				
**Total (95% CI)**			**100.0%**	**0.80 [0.66, 0.97]**
Heterogeneity: $Tau^2 = 0.03$; $Chi^2 = 22.77$, df = 4 (P = 0.0001); $I^2 = 82\%$				
Test for overall effect: Z = 2.32 (P = 0.02)				
Test for subgroup differences: $Chi^2 = 0.23$, df = 1 (P = 0.63), $I^2 = 0\%$				

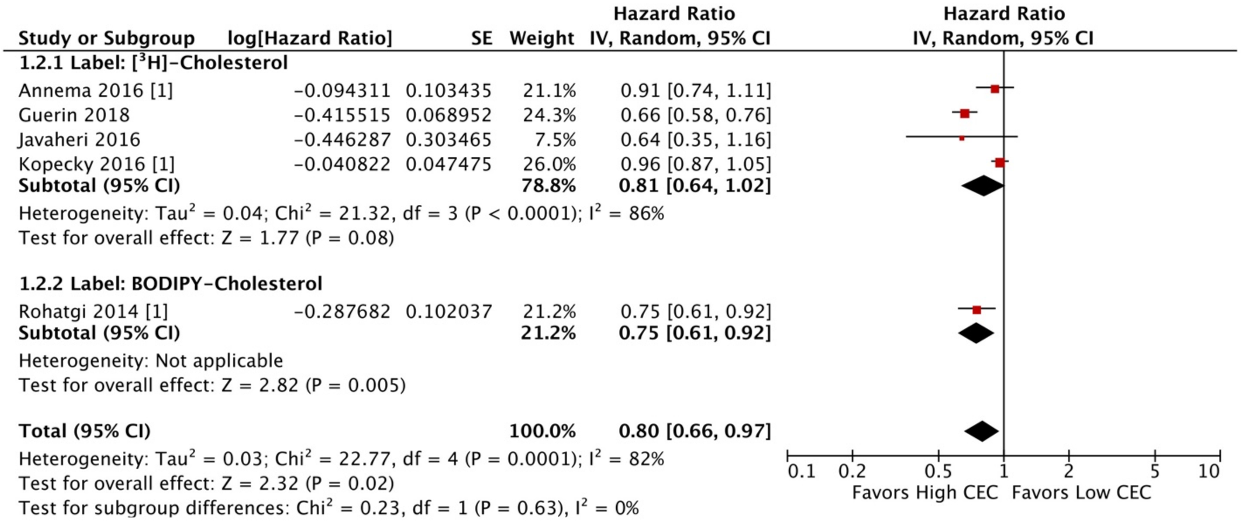

Figure S27. Adverse Cardiovascular Event Stratified by the Method of Labeling: Per SD Increment of CEC (adjusted HR) (10 studies)

Study or Subgroup	log[Hazard Ratio]	SE	Weight	Hazard Ratio IV, Random, 95% CI
**1.3.1 Label: [^{3}H]-Cholesterol**				
Annema 2016 [1]	−0.083382	0.168973	10.0%	0.92 [0.66, 1.28]
Guerin 2018	−0.235722	0.092914	12.8%	0.79 [0.66, 0.95]
Javaheri 2016	−1.347074	0.445214	3.5%	0.26 [0.11, 0.62]
Kopecky 2016 [1]	−0.020203	0.036505	14.3%	0.98 [0.91, 1.05]
**Subtotal (95% CI)**			**40.6%**	**0.83 [0.65, 1.05]**
Heterogeneity: Tau^2 = 0.04; Chi^2 = 13.14, df = 3 (P = 0.004); I^2 = 77%				
Test for overall effect: Z = 1.54 (P = 0.12)				
**1.3.2 Label: BODIPY-Cholesterol**				
Chindhy 2018 (CKD) [1]	0.262364	0.124239	11.7%	1.30 [1.02, 1.66]
Chindhy 2018 (Non-CKD) [1]	−0.223144	0.10474	12.4%	0.80 [0.65, 0.98]
Liu 2016 [1]	−2.302585	0.871869	1.1%	0.10 [0.02, 0.55]
Mody 2016 (FHx of MI)	−0.579818	0.173614	9.9%	0.56 [0.40, 0.79]
Mody 2016 (Overall)	−0.510826	0.117228	12.0%	0.60 [0.48, 0.75]
Rohatgi 2014 [1]	−0.385662	0.108032	12.3%	0.68 [0.55, 0.84]
**Subtotal (95% CI)**			**59.4%**	**0.72 [0.53, 0.96]**
Heterogeneity: Tau^2 = 0.10; Chi^2 = 32.54, df = 5 (P < 0.00001); I^2 = 85%				
Test for overall effect: Z = 2.22 (P = 0.03)				
**Total (95% CI)**			**100.0%**	**0.76 [0.63, 0.91]**
Heterogeneity: Tau^2 = 0.06; Chi^2 = 57.55, df = 9 (P < 0.00001); I^2 = 84%				
Test for overall effect: Z = 2.90 (P = 0.004)				
Test for subgroup differences: Chi^2 = 0.56, df = 1 (P = 0.45), I^2 = 0%				

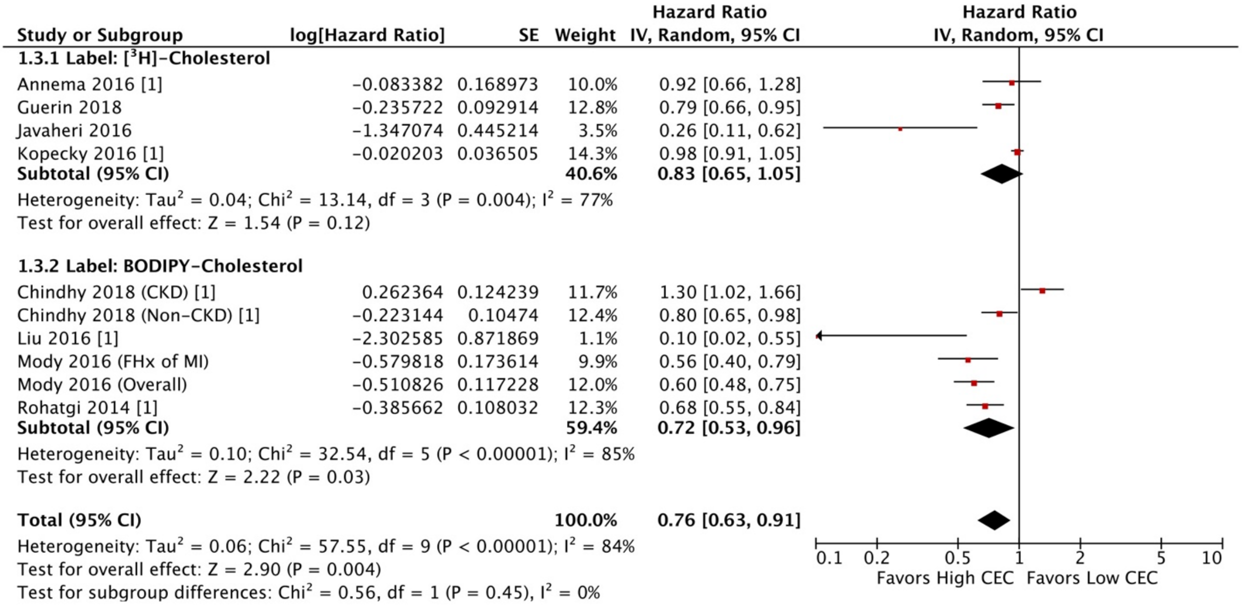

Supplement: Supplementary file 1 [file Data_Sheet_1.PDF]
